# Comprehensive characterization of endometrial competing endogenous RNA network in infertile women of childbearing age

**DOI:** 10.18632/aging.102874

**Published:** 2020-02-29

**Authors:** Meihua Zhang, Junxia Li, Shuyin Duan, Zhenya Fang, Jiaqi Tian, Haoyu Yin, Qingfeng Zhai, Xietong Wang, Lin Zhang

**Affiliations:** 1 Key Laboratory of Birth Regulation and Control Technology of National Health and Family Planning Commission of China, Maternal and Child Health Care Hospital of Shandong Province, Shandong University, Jinan 250001, China; 2 School of Public Health and Management, Weifang Medical University, Weifang 261042, China; 3 School of Public Health, Zhengzhou University, Zhengzhou 450001, China; 4 Department of Obstetrics and Gynecology, Shandong Provincial Hospital, Jinan 250001, China

**Keywords:** endometriosis, infertility, competing endogenous RNA, miRNA, lncRNA

## Abstract

Endometriosis is widely associated with infertility in women of childbearing age, for which there have been no effective treatments. Recent studies suggest that the dysregulation of RNAs contributes to the pathogenesis of endometriosis, so we conduct the case-control genetic analysis to characterize the expression and interaction of different subtypes of RNAs in infertile women with endometriosis. The ectopic and eutopic endometrium of patients undergoing infertility treatment were collected and subjected to high throughput sequencing, and bioinformatics analysis was conducted to construct the competing endogenous RNA (ceRNA) network. As a result, the RNA interactive network was constructed in endometriosis, and a set of mRNAs such as cyclin-dependent kinase 1 (CDK1) and proliferating cell nuclear antigen (PCNA) along with their corresponding miRNAs and lncRNAs were found to promote the growth and death of endometrial stromal cells, which was essential for the pathogenesis of endometriosis. These data suggest that RNA crosstalk is a crucial segment in the development of endometriosis, where CDK1 and PCNA may serve as emerging targets for the treatment of endometriosis-related infertility in women of childbearing age.

## INTRODUCTION

Endometriosis is characterized by the uterine tissue growing outside of the uterus, chronic inflammation of the endometrium, tissue-specific excess production of estrogen, and resistance to progesterone [1]. Worldwide, endometriosis affects 5-10% of women who are of reproductive age [2], and large-scale studies suggest a prevalence of 25 - 40% in infertile women, which is 5-8 times higher than that in fertile women [3]. For example, D’Hooghe and colleagues found that the morbidity of endometriosis was significantly higher in infertile women than that in fertile women, and infertile women were more likely to be diagnosed with advanced stages of endometriosis [4]. In particular cases, endometriosis is tightly associated with gynecologic cancer. Evidenced by previous studies, endometriosis increased the risk of ovarian cancer (standardized incidence ratio of 1.76 [95% confidence interval 1.47-4.38]) [5]. For a long time, various therapeutic strategies have been applied to endometriosis, but the expected yield was not achieved [6]. Among the most current therapeutic opinions for endometriosis-associated infertility, in vitro fertilization (IVF) is recommended with relatively high priority [7].

The pathogenesis of endometriosis is a complex biological process that is regulated by large amounts of molecules, in which messenger RNAs (mRNAs), microRNAs (miRNAs), and long noncoding RNAs (lncRNAs) are critical candidates. For mRNAs, anthrax toxin receptor 2 (ATR2) has been detected to be upregulated in endometriotic specimens, and elevation of ATR2 promotes endometriotic cell adhesion and angiogenesis, which ultimately leads to endometriosis [8]. Ubiquitin-specific protease 10 (USP10) acts similarly with ATR2, which promotes proliferation and migration of endometrial stromal cells via activating the Raf-1/MEK/ERK pathway [9]. miRNAs are dozens of highly conservative noncoding small RNAs that regulate mRNA translation and play essential roles in the pathogenesis of endometriosis. A meta-analysis based on 12 studies reported that 134 dysregulated miRNAs were potential biomarkers of endometriosis [10]. Specially, let-7 families were detected to be repressed in endometriosis, and loss of let-7 contributes to the development of endometriosis [11]. lncRNAs are kinds of noncoding/limited-coding RNAs with the length longer than 200 nucleotides [12], and aberrant expression of lncRNAs is indicated to be associated with endometrial hyperplasia [13]. MALAT1, a well-known lncRNA, could repress the expression of hypoxia-inducible factor-1α (HIF-1α), which exacerbated the autophagy of endometrial stromal cells and further promoted the pathogenesis of endometriosis [14].

In light of the crosstalk between different subtypes of RNAs, the competing endogenous RNA (ceRNA) network is proposed as different subtypes of RNAs competing for a limited pool of miRNAs [15]. In detail, miRNAs can repress the expression of their target genes or lncRNAs by targeting the miRNA response elements (MREs) on the 3′-untranslated region (UTR), coding sequence (CDS), or the 5′-UTR at the post-transcriptional level, and each miRNA can target numerous mRNAs or lncRNAs, while the vast majority of mRNAs or lncRNAs also harbor a certain number of MREs and are thus repressed by their corresponding miRNAs, which leads to the competition for the finite pool of miRNAs between lncRNAs and mRNAs [16, 17]. Fortunately, all ceRNA networks can be predicted using bioinformatics methods according to the principle of complementary base pairing. For example, the ceRNA network has been typically estimated in various physical disorders, including cancer, atherosclerosis, and central nervous system diseases, which provides insights for diseases in both clinical treatment and mechanism study [18–20]. In regards to the gynecologic disorders, lncRNA HOTAIR has been demonstrated to upregulate CCND1 and CCND2 by sponging miR-206 in ovarian cancer [21], and lncRNA MALAT1 could suppress tumor growth and progression through inhibiting PHF19 via sponging miR-211 [22]. Although many studies have suggested the indispensable role of ceRNA in various diseases, to the best of our knowledge, it has been seldom discussed in endometriosis.

To investigate the regulatory mechanism of different subtypes of RNAs in endometriosis, we collected the high throughput sequencing profiles of lncRNAs, miRNAs, and mRNAs to generate a new dataset, which was used to identify the differentially expressed RNAs (DERNAs) and construct the ceRNA network. The fitness of the ceRNA network was evaluated from 4 aspects of node degree, topological coefficient, closeness centrality, and betweenness centrality [23], which were all indicators of the social network analysis showing the relationship between RNAs. The biological function of the specific RNAs involved in the ceRNA network was enriched by using bioinformatics analysis through platforms of Gene Ontology (GO) and Kyoto Encyclopedia of Genes and Genomes (KEGG). The ceRNA network identified in this study would contribute to a better understanding of the pathogenesis of endometriosis-associated infertility and shed insights for etiological studies.

## RESULTS

### Characteristics of study subjects

The ectopic and eutopic endometrium from 14 infertile women with endometriosis were collected in this study, among whom the miRNAs and lncRNAs were profiled in 7 patients from the Chinese PLA general hospital, and the lncRNAs were detected in the rest 7 patients from the Fondazione Italiana Endometriosi. The median age of the patients used for miRNA/lncRNA profiling is lower than that of the mRNA group, but the difference was of no statistical significance (26 (25, 37) *vs.* 36 (34, 38), *P*>0.05). The distribution of the r-ASF stage between the ectopic and eutopic endometrium is similar (Table 1). These data indicate that samples involved in the combined dataset are of high homogeneity, which is comparable for the investigation of RNA interactions.

**Table 1:** Characteristics of 14 enrolled endometriosis patients from two datasets.

**Variables**	**mRNA/lncRNA**	**miRNA**	***Z/F* Value**	***P* Value**
N	7	7		
Age (yrs)*	26 (25, 37)	36 (34, 38)	-1.54	0.12
r-AFS stage			0.24	0.59
Phase III (%)	4 (57.14)	2 (28.57)		
Phase IV (%)	3 (42.86)	5 (71.43)		

*, Median (P25, P75).

### Identification of differentially expressed RNAs in endometriosis

**Figure 1:**
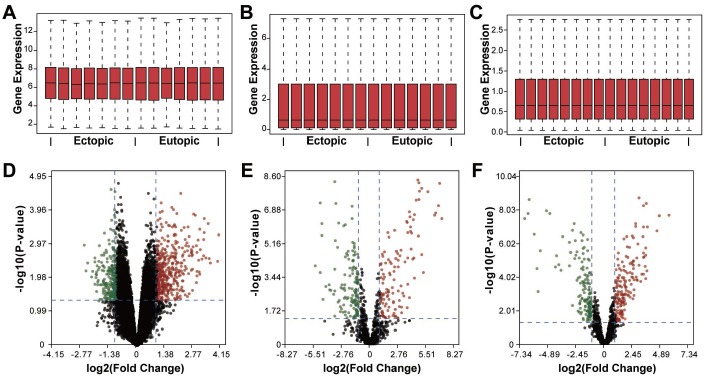
**Identification of differentially expressed RNAs in endometriosis.** (**A**–**C**) Box plots showing the expression of mRNAs, miRNAs, and lncRNAs. (**D**–**F**) Volcano plots of differentially expressed mRNAs, miRNAs, and lncRNAs. Red dots, upregulation, green dots, downregulation, black dots, rational expression.

**Figure 2:**
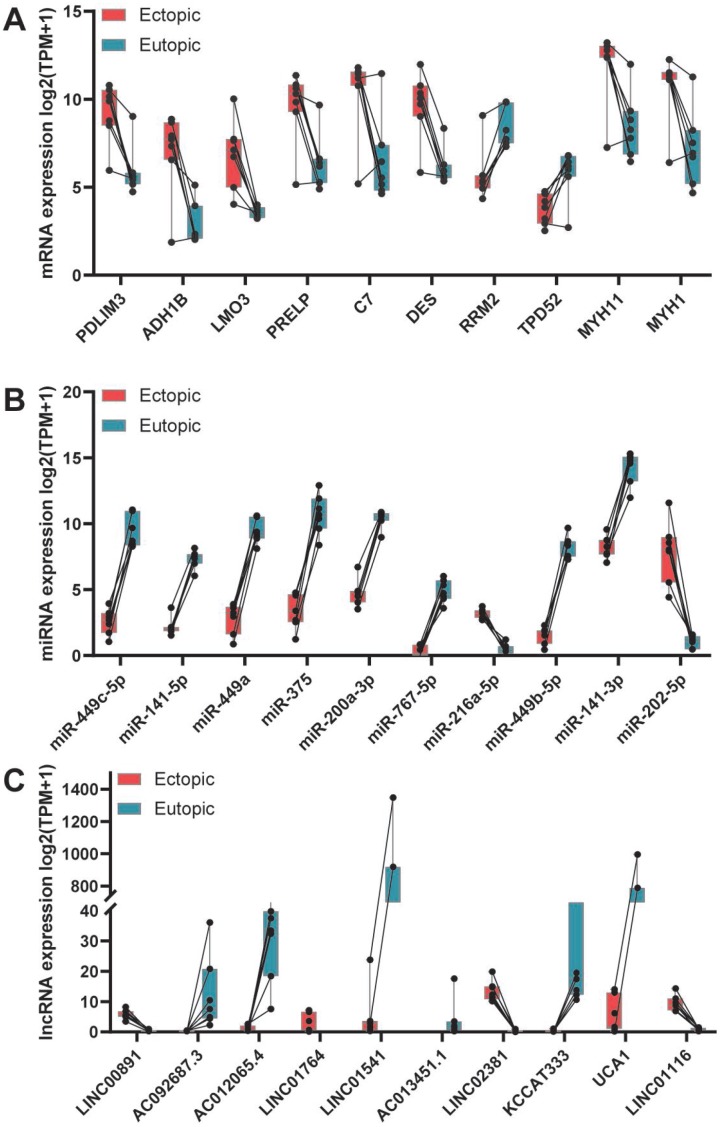
**Top 10 dysregulated RNAs in ectopic endometrium comparing with that in eutopic endometrium.** (**A**) Top 10 dysregulated mRNAs. (**B**) Top 10 dysregulated miRNAs. (**C**) Top 10 dysregulated lncRNAs.

To investigate the crosstalk between different subtypes of RNAs in endometriosis, we identified the dysregulated mRNAs, lncRNAs, and miRNAs, respectively. Three datasets regarding the genome-wide expression of mRNAs, lncRNAs, and miRNAs in the eutopic and ectopic endometrium were retrieved from the Gene Expression Omnibus (GEO): GSE105764, GSE105765, and GSE25628. In total, we found 22278 mRNAs, 20402 lncRNAs and 1014 miRNAs, among which 687 DEmRNAs, 263 DEmiRNAs, and 313 DElncRNAs were determined using limma analysis with rigorous data quality control (Figure 1), including 427 mRNAs, 161 lncRNAs, and 119 miRNAs that were upregulated, and 260 mRNAs, 152 lncRNAs, and 144 miRNAs that were downregulated. The top 10 dysregulated mRNAs, lncRNAs as well as miRNAs were visualized in Figure 2, and the full list of these RNAs could be referred to the Supplementary Tables 1–3.

### Construction of the lncRNA-miRNA-mRNA triple network in endometriosis

To explore the regulatory mechanism of RNA crosstalk in endometriosis, we constructed a triple network using miRNAs as a bridge between mRNAs and lncRNAs. In total, we identified 44290 mRNAs and 168170 lncRNAs that were targeted by the dysregulated miRNAs (Supplementary Figures 1 and 2). Furthermore, we extracted the DEmiRNAs that were associated with both mRNAs and lncRNAs to construct the lncRNA-miRNA-mRNA triple network, and, as a result, 9496 mRNAs, 11211 lncRNAs and 259 miRNAs were identified (Supplementary Figure 3).

**Figure 3:**
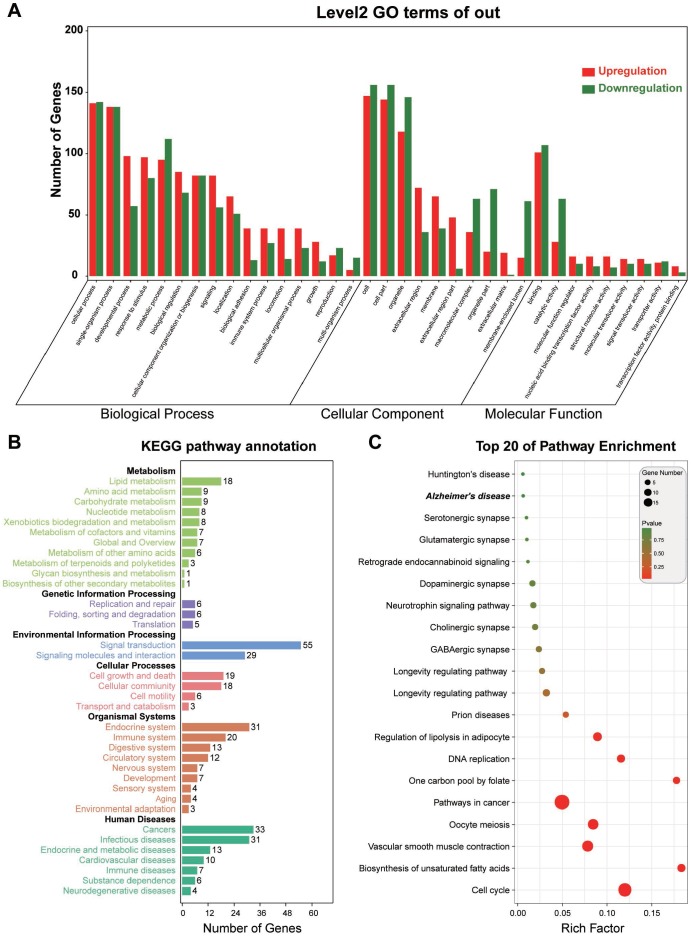
**Functional annotation for RNAs in the triple network.** (**A**) Gene Ontology analysis from 3 aspects of biological process, molecular function, and cellular components. (**B**) KEGG pathway annotation and enrichment. (**C**) Top 20 signaling pathways derived from KEGG signaling pathway enrichment.

To investigate the functional implication of the triple network, we adopted the functional annotation and enrichment analysis for RNAs involved in the triple network, and four aspects of biological process, molecular function, cellular component, and signaling pathway were investigated based on GO and KEGG. We found that the RNAs involved in the triple network were supposed to distribute in different organelles and play crucial roles in the developmental process, and these RNAs also regulate the catalytic activity and molecular function. The related signaling pathways are cell cycle and biosynthesis of unsaturated fatty acids (Figure 3). Thus, the RNAs in the triple network may contribute to the development of endometriosis through inducing disorders of cell cycle and metabolic processes.

### Identification of the ceRNA network in endometriosis

**Figure 4:**
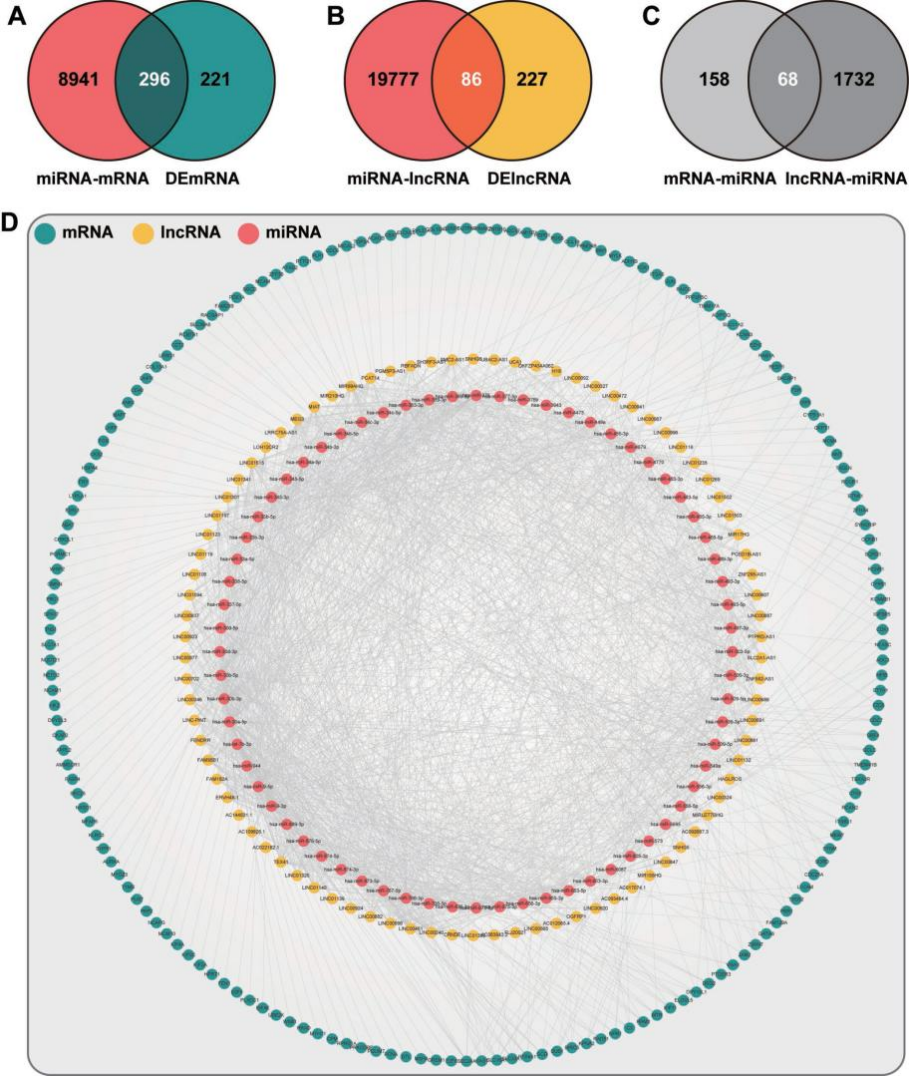
**The layout of the ceRNA network.** (**A**) Venn diagram of mRNAs. Red, mRNAs targeted by DEmiRNAs. Blue, mRNAs differentially expressed in endometriosis. (**B**) Venn diagram of lncRNAs. Red, lncRNAs targeted by DEmiRNAs. Yellow, lncRNAs differentially expressed in endometriosis. (**C**) Venn diagram of miRNAs. Light gray, miRNAs related to intersection mRNAs in (**A**). Dark gray, miRNAs related to intersection lncRNAs in (**B**). (**D**) The topological layout of ceRNA network based on DEmRNAs, DElncRNAs, and DEmiRNAs.

To identify the ceRNA network in endometriosis, we extracted the aberrantly expressed mRNAs and lncRNAs in the triple network. Utilizing the hypergeometric test, 172 DEmRNAs, 86 DElncRNAs, and 68 DEmiRNAs were determined, and 6871 DEmRNA-DElncRNA-DEmiRNA interactive items were identified in the ceRNA network (Figure 4).

### Fitness assessment and functional annotation of the ceRNA network

**Figure 5:**
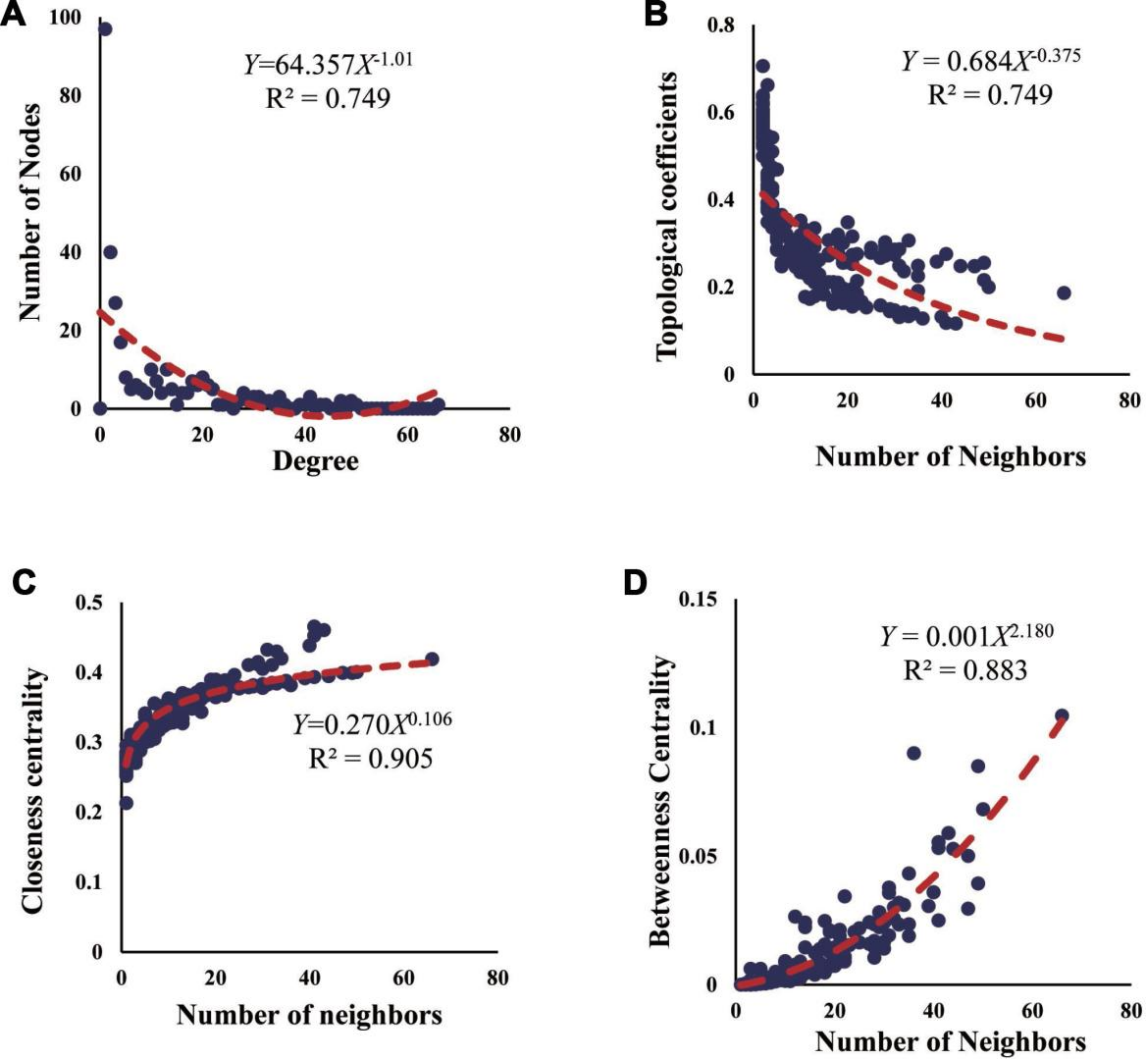
**Fitness assessment of the ceRNA network.** (**A**–**D**) Assessment of ceRNA network from 4 aspects of node degree, topological coefficient, closeness centrality, and betweenness centrality.

To explore the fitness of the ceRNA network, we conducted the topological assessment from 4 aspects of node degree, topological coefficient, closeness centrality, and betweenness centrality (Figure 5). As previously indicated, the node degree reflects the possibility for a node to be targeted by other nodes, the topological coefficient explains the number of neighbors shared by a pair of nodes, the closeness centrality implies the potential for a node to be a center, and the betweenness centrality suggests the role for a node in connecting different modules [23]. Herein, the R squares of node degree, topological coefficient, closeness centrality, and betweenness centrality are 0.749, 0.749, 0.905, and 0.883, indicating the high efficiency of the ceRNA network.

**Figure 6:**
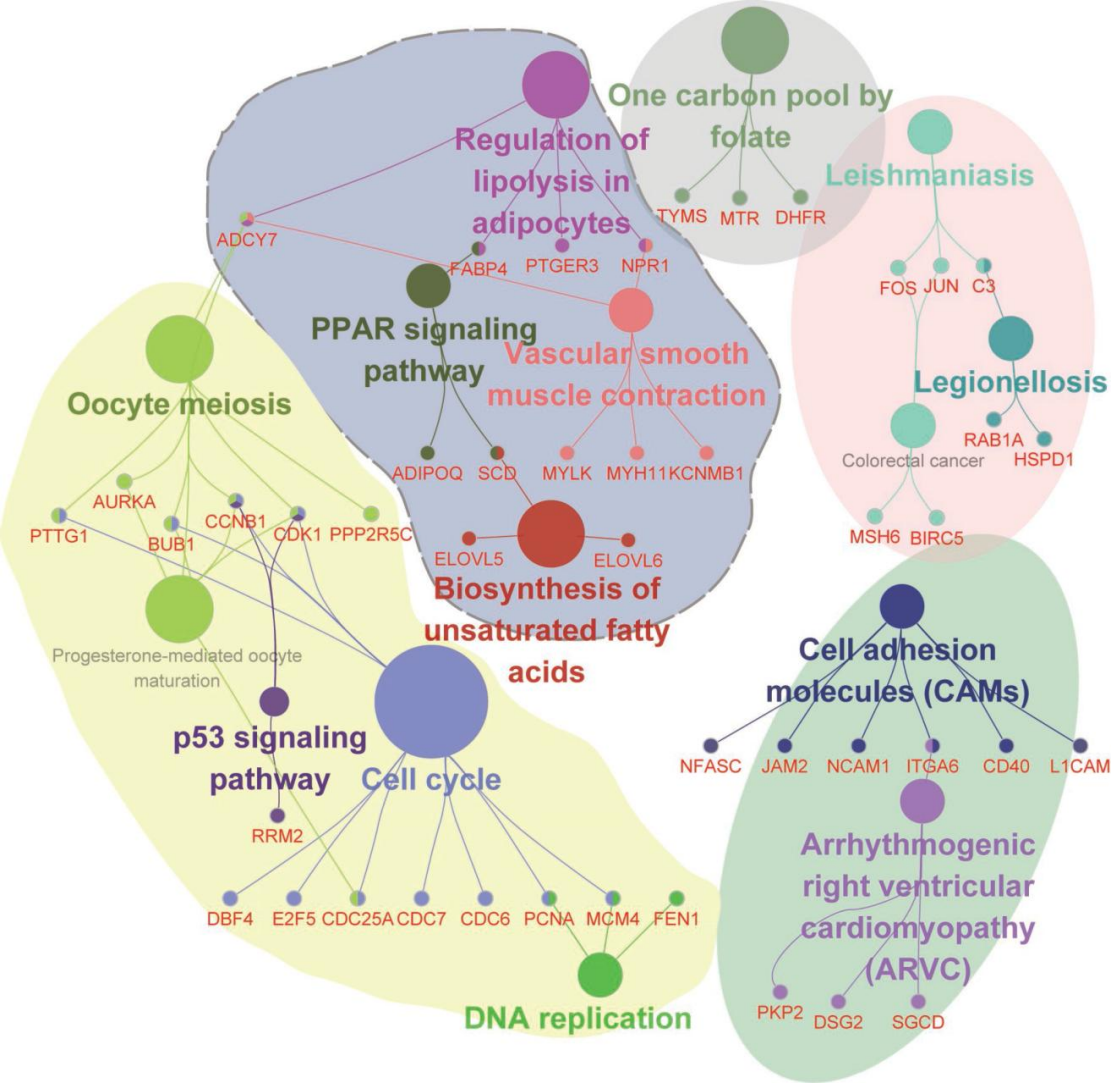
**Functional enrichment analysis for RNAs involved in the ceRNA network.** Functional enrichment modules are represented in different colors.

Additionally, we adopted functional annotation for RNAs in the ceRNA network (Figure 6), and five functional groups were identified, including the p53 signaling pathway, cell cycle, DNA replication, PPAR signaling pathway, and biosynthesis of unsaturated fatty acids. Importantly, a set of hub genes such as PTTG1, BUB1, AURKA, CCNB1, CDK1, PPP2R5C, and CDC25A were determined as crucial regulators of cell proliferation and oocyte maturation, which contributed to the maintenance of menstrual cycle. Therefore, these data demonstrate that the ceRNA network constructed in this study is of high accuracy in exploring the mechanism of endometriosis, and the critical regulators identified in the ceRNA network are potential biomarkers for the diagnosis of endometriosis.

## DISCUSSION

Endometriosis is one of the most common gynecologic diseases with high risk for infertility and gynecologic cancer, particularly in patients who underwent surgical operation of the uterus [24]. However, the pathogenesis of endometriosis is not fully clarified. In general, it is widely accepted that endometriosis could be induced by various factors, and pelvic inflammation has been identified as a prerequisite for the occurrence of endometriosis [25]. With the development of transcriptomics, recent studies confirmed the essential role of different subtypes of RNAs in endometriosis, and a set of well-studied RNAs, including mRNAs, lncRNAs, and miRNAs, had been identified as crucial regulators of endometriosis. For example, NLRC5 and P62 were significantly upregulated in the ectopic endometrium, while the elevation of NLRC5 corresponded to autophagy inhibition in endometriosis, which could be used as indicators for the diagnosis of endometriosis [26]. As a transcriptional regulator, circulating miR-370-3p was demonstrated to repress cell proliferation by downregulating steroidogenic factor 1 (SF1) in endometriosis [27]. CDK6 was found to upregulate the expression of lncRNA AC002454.1 and further accelerate cell cycle, which promoted the progression of endometriosis [28]. Besides, our previous work also showed an indispensable role of let-7 families in promoting nanoparticle phagocytosis in lung cancer [29–31]. Thus, it is necessary to grasp the RNA characteristics at the genome-wide level, which will provide evidence for the mechanism study as well as the clinical treatment of endometriosis.

Using publicly available datasets, we recombined a new dataset containing the expression profiles of mRNAs, lncRNAs, and miRNAs. Then, we identified the dysregulated RNAs in the ectopic endometrium comparing with that in the eutopic endometrium. In total, 687 DEmRNAs, 263 DEmiRNAs, and 313 DElncRNAs were determined, among which 427 mRNAs, 161 lncRNAs, and 119 miRNAs were upregulated, and 260 mRNAs, 152 lncRNAs, and 144 miRNAs were downregulated. Of the 687 DEmRNAs, the top 10 mRNAs, including LMO3, DES, ADH1B, PDLIM3, ADH7, RRM2, PRELP, TPD52, MYH11, and MYH1, were shown in Figure 2. Interestingly, PRELP has been demonstrated to be upregulated in patients with endometriosis [32]. Meanwhile, most of the top 10 DEmiRNAs have also been validated in endometriosis [33]. miR-202, for example, was found to promote endometriosis by inhibiting SOX6 [34], and downregulation of miR-375 alleviated the suppression of endothelin 1 (EDN1) in ectopic stromal cells, which in turn contributed to the development of endometriosis [35]. Moreover, direct transfection of miR-449b-3p into endometrial stromal cells inhibited the proliferation and formation of tubular structures in human umbilical vein endothelial cells [36]. For miR-216a-5p and miR-767-5p, available studies concerning their roles in endometriosis were not found. Similarly, there are no studies concerning the top 10 dysregulated lncRNAs in endometriosis, but the DElncRNA H19 has been reported as an efficient regulator in altering stromal cell growth via IGF signaling pathway in the endometriosis [37].

Furthermore, we constructed a triple network and performed functional annotation for RNAs in the triple network. As shown in Figure 4, signaling pathways of cell cycle and DNA replication (KEGG id: ko04110 and ko03030) were determined with high-risk factors, gene numbers, and shallow *P* values. Consistent with previous studies, dysregulation of cell cycle and DNA replication adversely affected the proliferation of endometrial stromal cells, and several dysregulated genes such as CDC2, PCNA, and CCN1 have also been previously investigated [38]. Specially, PCNA was crucial in the maintenance of cell proliferation, and downregulation of PCNA promoted the proliferation and invasion of endometrial stromal cells [39]. CCN1 was a secretory cysteine-rich matricellular protein that highly expressed in human ectopic endometriotic lesions, as a regulator of estrogen at downstream, CCN1 controled cell proliferation and neovascularization [40]. However, Tang L and colleagues found that there was no difference in CDC2 expression between ectopic and eutopic endometrium [41], so further studies are still needed to confirm the regulatory mechanism of CDC2 in endometriosis.

For the downstream investigation, we constructed the ceRNA network based on the RNA triple network. A total of 6871 DEmRNA-DElncRNA-DEmiRNA interactive items were identified, but only two studies concerning the ceRNA regulatory mechanism in endometriosis were found, which all focused on the circRNA-related ceRNA network [42, 43], so it was hard to provide convincing evidence from previous studies for the putative ceRNA network. Indeed, the RNAs in the ceRNA network have mostly been demonstrated to be associated with endometriosis, and the biological processes concluded from the functional annotation were also highly consistent with the pathogenesis of endometriosis. Cell adhesion molecules (CAM), for example, were a kind of cell surface transmembrane glycoproteins that promoted cell-to-cell adhesion and directly affect cell proliferation, differentiation, extension, and migration, which were essential for the occurrence and development of endometriosis [44]. As a bridge connecting the PPAR signaling pathway and the lipolysis in adipocytes signaling pathway, FABP4 had been demonstrated to manipulate the metabolism and differentiation of endometrial stromal cells [45], so the circulating fatty acid level may influence the pathological procedure of endometriosis. Consistently, the folate and the biosynthesis of unsaturated fatty acids have been identified in the functional enrichment analysis, which could be linked to endometriosis development. Also, the P53 signaling pathway acted as a guardian in tumor suppression [46], where three relevant genes were identified in endometriosis and maybe ligaments for studying the relationship between endometriosis and endometrial cancer. At last, we conducted the fitness assessment for the ceRNA network. As indicated by R squares of the number of nodes, betweenness centrality, closeness centrality, and topological coefficient, the ceRNA network was of high efficiency and could be used for guiding future studies.

Since this is cross-sectional research, which does limit the causal relationship proposed in this study. Moreover, a total of 14 patients were recruited to construct the ceRNA network, and this may lead to Type II errors due to the limited sample size. Although several interactions of the ceRNA network have been determined in previous studies, the ceRNA network is still needed to be verified using further experiments either *in vitro* or *in vivo*. Importantly, several factors, including the calculating parameters and software versions, may influence the reproducibility of the ceRNA network and should be strictly set according to our descriptions in future studies

To sum up, we construct a ceRNA network with high efficiency and precision based on the genome-wide expression profiles of mRNAs, lncRNAs, and miRNAs in endometriosis. The crosstalk between RNAs of different subtypes, including CDK1, CCNB1, E2F5, and PCNA along with their corresponding miRNAs and lncRNAs, is speculated to promote the development of endometriosis, which provides insights and perspectives for the investigation of endometriosis-associated infertility.

## MATERIALS AND METHODS

### Patients

A total of 14 infertile women with endometriosis were enrolled in this study. All patients were confirmed by histological examination and diagnosed with endometriosis at moderate to severe stage using the revised American Fertility Society (r-AFS) classification standards [47, 48], women in the secretory phase of the menstrual cycle and without any hormonal treatment history were included in the study. The clinical samples of the eutopic or ectopic ovarian endometrial tissues were collected from women who underwent surgery for infertility, pelvic pain symptoms, or adnexal masses during laparoscopy surgery or curettage before the laparoscopic procedure, the baseline characteristics including age and r-AFS stage were also listed in Table 1. A total of 7 out of the 14 patients from Rome offered samples for mRNA detection, and the remaining patients from China at the Chinese PLA general hospital denoted samples for miRNA/lncRNA sequencing. All patients were well informed and admitted about the purpose of this study.

### Data quality control and dataset recombination

The high throughput sequencing profiles of miRNAs (GSE105764) and lncRNAs (GSE105764), as well as the mRNA microarray dataset (GSE25628), were retrieved from GEO publicly available at https://www.ncbi.nlm.nih.gov/geo/. All data were normalized using the quantile normalization method, in which RNAs with missing values were excluded. The homogeneity of all samples was checked using cluster analysis and principal component analysis, where heterogeneously distributed samples were removed.

### Identification of differentially expressed RNAs

The expression patterns of mRNAs, lncRNAs and miRNAs were compared using bioinformatics analysis through R software version 3.4.1 for windows, and the DERNAs were identified using the following criteria: limma comparison method, *P*-value < 0.05, and the absolute value of (log_2_(fold change)) ≥ 1. The differentially expressed RNAs were summarized and used for the construction of the ceRNA network.

### Construction of the mRNA-miRNA-lncRNA triple network

Firstly, the putative regulatory patterns of miRNA-lncRNA and miRNA-mRNA were determined according to the principle of complementary base pairing. Using miRNA as a bridge, we constructed a triple network among miRNAs, lncRNAs, and mRNAs. In detail, the mRNAs targeted by the differentially expressed miRNAs were predicted using microT scoring method (paring score ≥ 0.8) via the DIANA-microT-CDS plugin in DIANA platform (available online at http://www.microrna.gr/microT-CDS) [49], and the lncRNAs repressed by the dysregulated miRNAs were identified through the lncBase database version 2.0, a database elaborated information for predicted and experimental verified miRNA-lncRNA interactions [50]. Finally, the mRNA-miRNA-lncRNA interactions were visualized using a topological network in Cytoscape version 3.7.1.

### Visualization and fitness assessment of the ceRNA network

To identify the ceRNA network, we extracted the DEmRNAs and DElncRNAs in the triple network, and we used the hypergeometric test to evaluate the significance of the shared miRNAs between each mRNA and lncRNA, a *P*-value < 0.05 was considered as statistically significant, and it was calculated as follows:

$$P=1-\sum_{t=0}^{x}\frac{\left(\begin{array}{c} t\\ K \end{array}\right)\left(\begin{array}{c} N-t\\ M-K \end{array}\right)}{\left(\begin{array}{c} N\\ M \end{array}\right)}$$

Where K is the number of miRNAs interacted with mRNAs, N represents the number of miRNAs interacted with lncRNAs, M is the number of miRNAs in the genome, *x* is the number of miRNAs shared by mRNAs and lncRNAs [51].

Fitness assessment of the ceRNA network was performed using the NetworkAnalyzer plugin in Cytoscape version 3.7.1 [23], several properties including the degree of node, topological coefficient, betweenness centrality, and closeness centrality were used to investigate the efficiency of the model. The specific calculation methods could be referred to the manual of SocNetV V2.0, a software for visualizing social networks.

### Functional annotation and enrichment analysis for differentially expressed RNAs

Functional enrichment analysis was performed from 4 aspects of biological process, molecular function, cellular component, and signaling pathway using GO and KEGG database through ClueGO plugin in Cytoscape version 3.7.1 [52], the analytical parameters were set as default: two-side hypergeometric test, P-value of pathway enrichment ≤ 0.05, Kappa score of GO term/pathway network connectivity ≥ 0.4, GO tree interval between 3 and 8, and the number of genes involved in each cluster ≥ 3. For cluster analysis of different functional modules, we used EnrichmentMap plugin in Cytoscape Version 3.7.1 [53], which was used to show the pathways as an enrichment map and further highlight the overlaps among different pathways. GO and KEGG were available online at http://geneontology.org/ and https://www.kegg.jp/, respectively.

## Supplementary Material

Supplementary Figures

Supplementary Table 1

Supplementary Table 2

Supplementary Table 3
