# Supplementary material for: Comprehensive characterization of endometrial competing endogenous RNA network in infertile women of childbearing age
Source: Aging (Albany NY). 2020 Feb 29;12(5):4204–21. doi: 10.18632/aging.102874 (PMC7093184; doi:10.18632/aging.102874)
Supplement: Supplementary Figures [file aging-12-102874-s004..pdf]

SUPPLEMENTARY FIGURES

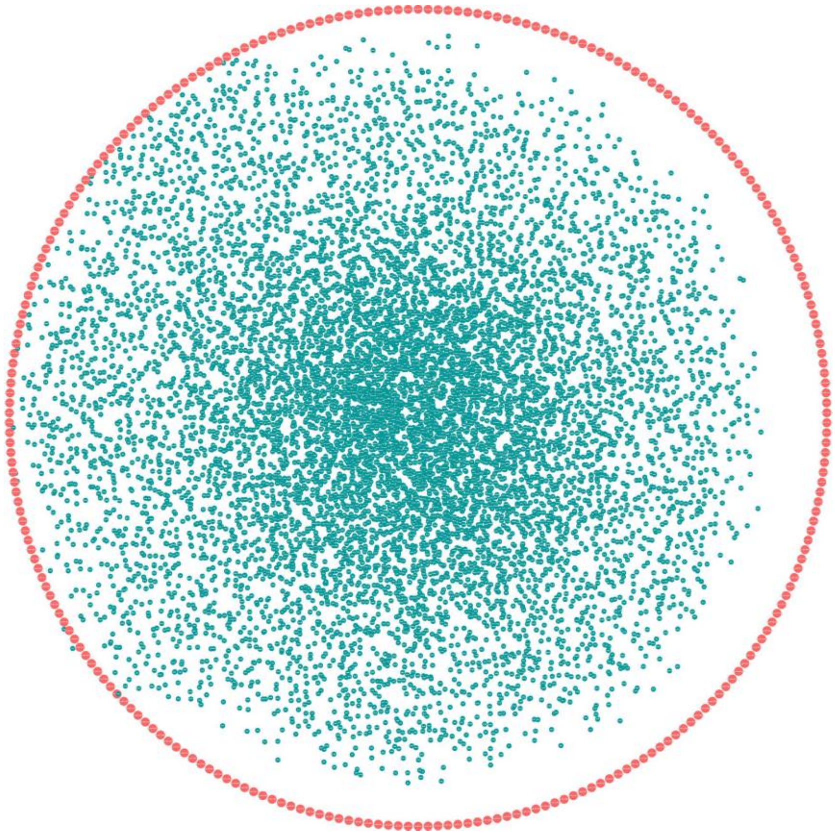

Supplementary Figure 1. Target genes of the differentially expressed miRNAs in endometriosis. Pink dots, miRNA, blue dots, mRNA.

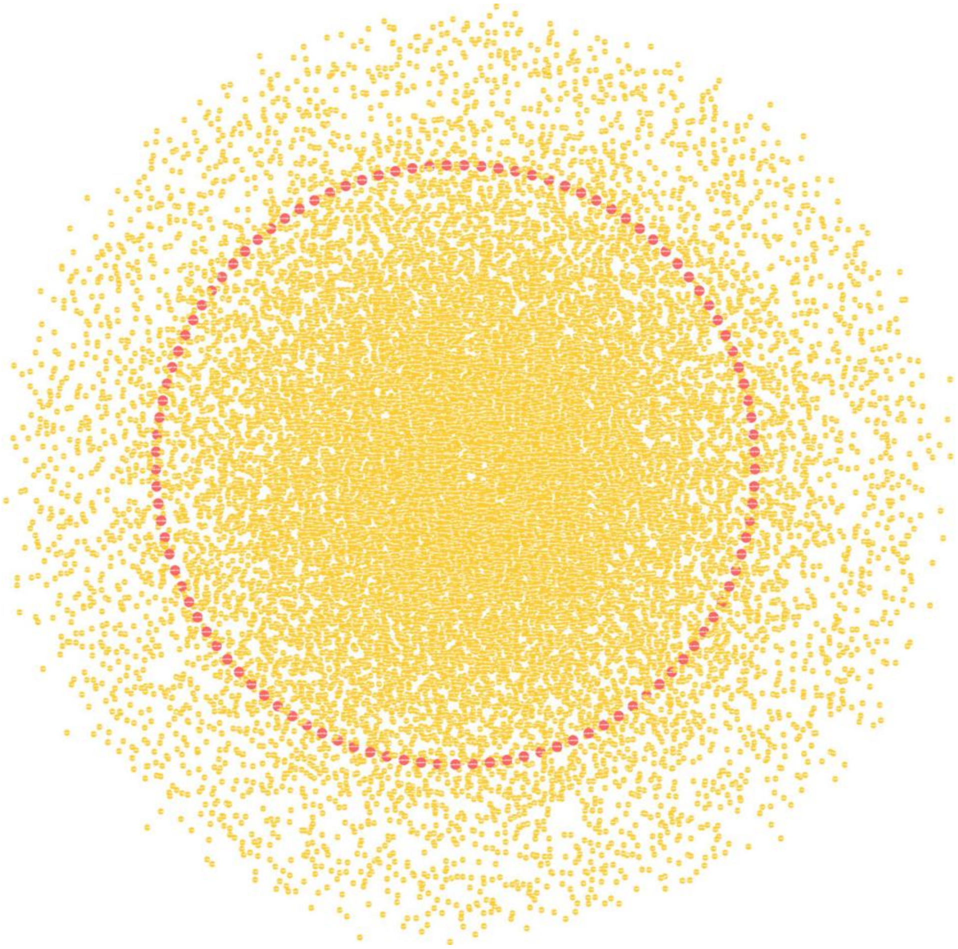

Supplementary Figure 2. Target lncRNAs of the differentially expressed miRNAs in endometriosis. Pink dots, miRNA, yellow dots, lncRNAs.

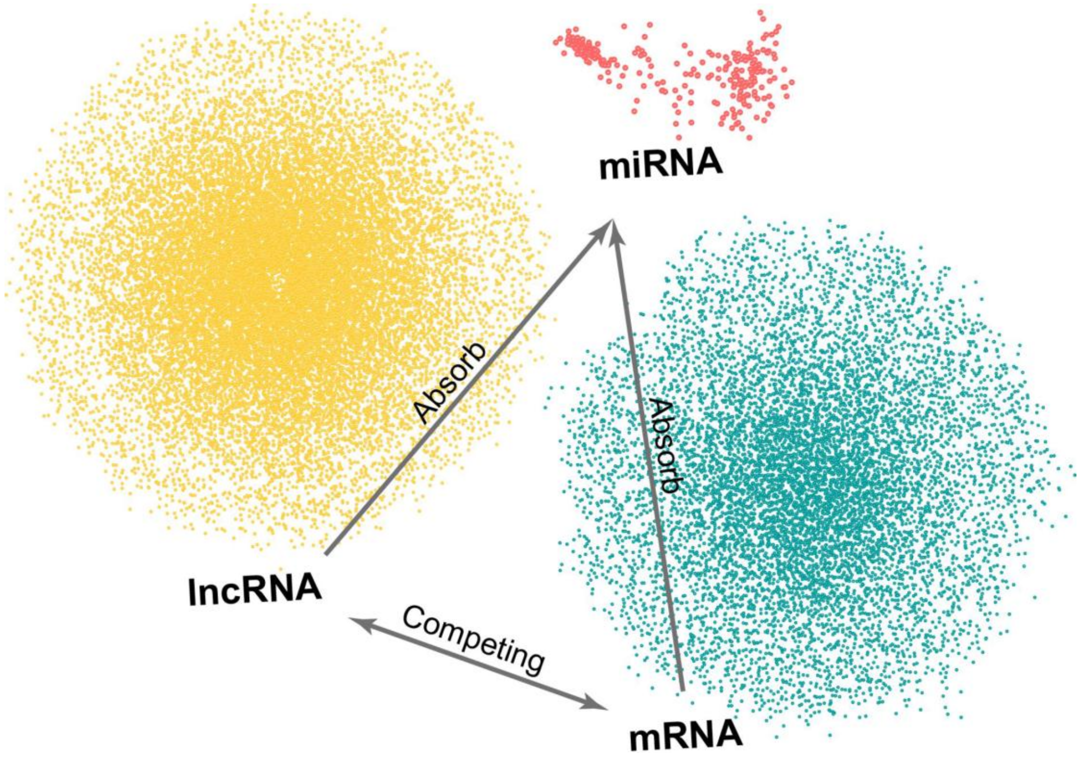

Supplementary Figure 3. Triple network of mRNA-miRNA-lncRNA in endometriosis. Pink dots, miRNA, yellow dots, lncRNAs, blue dots, mRNA.
