# Supplementary material for: Comprehensive characterization of endometrial competing endogenous RNA network in infertile women of childbearing age
Source: Aging (Albany NY). 2020 Feb 29;12(5):4204–21. doi: 10.18632/aging.102874 (PMC7093184; doi:10.18632/aging.102874)
Supplement: Supplementary Table 1 [file aging-12-102874-s003..docx]

**Supplementary Table 1. Full list of differentially expressed Genes in Ectopic Endometrium.**

| **ID** | **Gene_Symbol** | **log_2_(Fold Change)** | **Regulation** | **t Value** | **B Value** | **P Value** |
| --- | --- | --- | --- | --- | --- | --- |
| 1 | ERMP1 | -1.25 | Down | -6.46 | 3.22 | 1.11E-05 |
| 2 | CYP51A1 | -1.26 | Down | -5.99 | 2.54 | 2.56E-05 |
| 3 | SRR | -1.08 | Down | -5.9 | 2.42 | 2.98E-05 |
| 4 | PLVAP | 2.18 | Up | 5.83 | 2.32 | 3.37E-05 |
| 5 | MYOM1 | 1.21 | Up | 5.5 | 1.8 | 6.28E-05 |
| 6 | L1CAM | 1.72 | Up | 5.33 | 1.54 | 8.61E-05 |
| 7 | VSNL1 | 1.81 | Up | 5.26 | 1.42 | 9.88E-05 |
| 8 | SEC24A | -1.09 | Down | -5.24 | 1.4 | 1.01E-04 |
| 9 | GAS6 | 1.27 | Up | 5.2 | 1.33 | 1.10E-04 |
| 10 | SLC24A1 | -1.02 | Down | -5.18 | 1.3 | 1.14E-04 |
| 11 | MEGF6 | 1.14 | Up | 5.15 | 1.24 | 1.22E-04 |
| 12 | MGP | 1.98 | Up | 5.12 | 1.2 | 1.29E-04 |
| 13 | SCRG1 | 2.82 | Up | 5.1 | 1.16 | 1.34E-04 |
| 14 | CLU | 2.88 | Up | 5.05 | 1.09 | 1.45E-04 |
| 15 | SLC33A1 | -1.09 | Down | -5.05 | 1.09 | 1.46E-04 |
| 16 | PTP4A1 | -1.07 | Down | -4.97 | 0.95 | 1.71E-04 |
| 17 | CXCL6 | 1.14 | Up | 4.94 | 0.9 | 1.82E-04 |
| 18 | LMO3 | 3.33 | Up | 4.93 | 0.9 | 1.83E-04 |
| 19 | ELOVL5 | -1.23 | Down | -4.88 | 0.81 | 2.02E-04 |
| 20 | ADH1B | 1.04 | Up | 4.87 | 0.79 | 2.07E-04 |
| 21 | ZBED4 | -1.02 | Down | -4.79 | 0.66 | 2.43E-04 |
| 22 | DES | 3.54 | Up | 4.76 | 0.61 | 2.55E-04 |
| 23 | GLT25D2 | 1.19 | Up | 4.73 | 0.56 | 2.71E-04 |
| 24 | ITGA7 | 1.86 | Up | 4.72 | 0.54 | 2.79E-04 |
| 25 | MMP28 | 1.62 | Up | 4.68 | 0.46 | 3.03E-04 |
| 26 | MEOX1 | 1.84 | Up | 4.67 | 0.46 | 3.04E-04 |
| 27 | CASQ2 | 1.91 | Up | 4.64 | 0.41 | 3.23E-04 |
| 28 | PTGIS | 1.25 | Up | 4.64 | 0.4 | 3.27E-04 |
| 29 | HSPB8 | 1.75 | Up | 4.64 | 0.4 | 3.28E-04 |
| 30 | SELP | 2.33 | Up | 4.62 | 0.36 | 3.41E-04 |
| 31 | DLAT | -1.29 | Down | -4.57 | 0.28 | 3.74E-04 |
| 32 | CNN1 | 3.12 | Up | 4.56 | 0.27 | 3.79E-04 |
| 33 | HSPB7 | 2.21 | Up | 4.53 | 0.21 | 4.07E-04 |
| 34 | SOAT1 | -1.17 | Down | -4.53 | 0.21 | 4.08E-04 |
| 35 | SPEG | 1.18 | Up | 4.52 | 0.21 | 4.09E-04 |
| 36 | HSPA4 | -1.06 | Down | -4.46 | 0.1 | 4.61E-04 |
| 37 | PDLIM3 | 3.33 | Up | 4.45 | 0.07 | 4.77E-04 |
| 38 | PKP2 | -1.58 | Down | -4.43 | 0.05 | 4.91E-04 |
| 39 | PHTF1 | -1.03 | Down | -4.42 | 0.03 | 5.03E-04 |
| 40 | PTPRG | -1.04 | Down | -4.41 | 0.01 | 5.15E-04 |
| 41 | KCNN3 | 1.88 | Up | 4.39 | -0.01 | 5.29E-04 |
| 42 | FIGF | 1.92 | Up | 4.38 | -0.05 | 5.48E-04 |
| 43 | PDE1A | 1.84 | Up | 4.35 | -0.1 | 5.82E-04 |
| 44 | LRRN3 | 1.54 | Up | 4.33 | -0.12 | 5.99E-04 |
| 45 | FZD7 | 2.59 | Up | 4.31 | -0.16 | 6.28E-04 |
| 46 | UBE2K | -1.01 | Down | -4.3 | -0.18 | 6.41E-04 |
| 47 | ACACB | 1.25 | Up | 4.27 | -0.22 | 6.73E-04 |
| 48 | EPHA3 | 2.4 | Up | 4.22 | -0.31 | 7.46E-04 |
| 49 | KCNMB1 | 3.29 | Up | 4.21 | -0.33 | 7.59E-04 |
| 50 | CPEB1 | 1.37 | Up | 4.21 | -0.34 | 7.68E-04 |
| 51 | NNT | -1.37 | Down | -4.21 | -0.34 | 7.69E-04 |
| 52 | CHRDL1 | 3.04 | Up | 4.2 | -0.35 | 7.80E-04 |
| 53 | CSPG4 | 1 | Up | 4.19 | -0.37 | 8.01E-04 |
| 54 | LDB3 | 1.92 | Up | 4.17 | -0.4 | 8.24E-04 |
| 55 | GINS3 | -1.19 | Down | -4.17 | -0.4 | 8.29E-04 |
| 56 | ARHGEF4 | 1.57 | Up | 4.16 | -0.43 | 8.50E-04 |
| 57 | SRPX | 1.54 | Up | 4.16 | -0.43 | 8.51E-04 |
| 58 | SLITRK3 | 2 | Up | 4.13 | -0.48 | 9.05E-04 |
| 59 | PTGER3 | 2.49 | Up | 4.1 | -0.52 | 9.49E-04 |
| 60 | APPL2 | -1.04 | Down | -4.1 | -0.53 | 9.54E-04 |
| 61 | MCTP1 | 1.2 | Up | 4.07 | -0.58 | 1.01E-03 |
| 62 | RRM2 | -2.6 | Down | -4.03 | -0.65 | 1.10E-03 |
| 63 | SGCG | 1.76 | Up | 4.03 | -0.65 | 1.11E-03 |
| 64 | HMGCR | -1.83 | Down | -4.02 | -0.67 | 1.13E-03 |
| 65 | MYOT | 1.06 | Up | 4.01 | -0.69 | 1.15E-03 |
| 66 | DPT | 2.43 | Up | 4.01 | -0.69 | 1.15E-03 |
| 67 | PLN | 2.95 | Up | 4.01 | -0.69 | 1.15E-03 |
| 68 | PTGFR | 1.52 | Up | 3.99 | -0.72 | 1.19E-03 |
| 69 | DARC | 2.77 | Up | 3.98 | -0.74 | 1.22E-03 |
| 70 | CADM3 | 2.09 | Up | 3.96 | -0.77 | 1.26E-03 |
| 71 | HPRT1 | -1.17 | Down | -3.96 | -0.78 | 1.28E-03 |
| 72 | NRN1 | 1.8 | Up | 3.95 | -0.79 | 1.29E-03 |
| 73 | HOXA7 | 1.02 | Up | 3.95 | -0.79 | 1.30E-03 |
| 74 | AGTR1 | 2.25 | Up | 3.93 | -0.82 | 1.34E-03 |
| 75 | WSB1 | 1.28 | Up | 3.93 | -0.83 | 1.35E-03 |
| 76 | PDE2A | 1.88 | Up | 3.91 | -0.87 | 1.41E-03 |
| 77 | SLCO2A1 | 1.41 | Up | 3.89 | -0.89 | 1.46E-03 |
| 78 | WIF1 | 3.14 | Up | 3.89 | -0.9 | 1.47E-03 |
| 79 | TIMM17A | -1.14 | Down | -3.88 | -0.92 | 1.49E-03 |
| 80 | KCNA5 | 1.4 | Up | 3.88 | -0.92 | 1.49E-03 |
| 81 | NINJ2 | 1.02 | Up | 3.88 | -0.92 | 1.49E-03 |
| 82 | RCOR1 | -1.13 | Down | -3.88 | -0.92 | 1.51E-03 |
| 83 | SNX24 | -1.02 | Down | -3.87 | -0.94 | 1.53E-03 |
| 84 | MN1 | 1.91 | Up | 3.86 | -0.96 | 1.56E-03 |
| 85 | SQLE | -1.5 | Down | -3.86 | -0.96 | 1.56E-03 |
| 86 | ITIH5 | 1.22 | Up | 3.85 | -0.97 | 1.60E-03 |
| 87 | ATP2A3 | 1.03 | Up | 3.85 | -0.98 | 1.60E-03 |
| 88 | MEOX2 | 2.5 | Up | 3.85 | -0.98 | 1.60E-03 |
| 89 | VWA1 | 1.03 | Up | 3.84 | -0.99 | 1.63E-03 |
| 90 | RPRD1A | -1.08 | Down | -3.81 | -1.05 | 1.74E-03 |
| 91 | SORBS1 | 2.36 | Up | 3.8 | -1.05 | 1.75E-03 |
| 92 | CSRP1 | 1.45 | Up | 3.8 | -1.06 | 1.77E-03 |
| 93 | KPNA2 | -1.49 | Down | -3.79 | -1.08 | 1.79E-03 |
| 94 | MYH11 | 2.96 | Up | 3.79 | -1.08 | 1.79E-03 |
| 95 | TCEAL2 | 2.7 | Up | 3.78 | -1.09 | 1.82E-03 |
| 96 | LIMS2 | 1.83 | Up | 3.78 | -1.1 | 1.83E-03 |
| 97 | CX3CL1 | 1.19 | Up | 3.78 | -1.1 | 1.83E-03 |
| 98 | DHFR | -1.58 | Down | -3.77 | -1.11 | 1.86E-03 |
| 99 | FAM129A | 1.73 | Up | 3.77 | -1.11 | 1.87E-03 |
| 100 | FXYD1 | 1.96 | Up | 3.77 | -1.12 | 1.88E-03 |
| 101 | JAM2 | 1.28 | Up | 3.76 | -1.12 | 1.90E-03 |
| 102 | GADD45B | 1.37 | Up | 3.76 | -1.14 | 1.93E-03 |
| 103 | LPP | 1.05 | Up | 3.75 | -1.15 | 1.95E-03 |
| 104 | CPE | 1.93 | Up | 3.72 | -1.2 | 2.07E-03 |
| 105 | ENO2 | 1.07 | Up | 3.72 | -1.2 | 2.07E-03 |
| 106 | AOC3 | 2.75 | Up | 3.71 | -1.21 | 2.10E-03 |
| 107 | CCL19 | 2.29 | Up | 3.71 | -1.22 | 2.11E-03 |
| 108 | SEC23B | -1.03 | Down | -3.71 | -1.23 | 2.13E-03 |
| 109 | MCM9 | -1.11 | Down | -3.71 | -1.23 | 2.13E-03 |
| 110 | RBM47 | -1.72 | Down | -3.69 | -1.26 | 2.20E-03 |
| 111 | TMEM41B | -1.09 | Down | -3.69 | -1.26 | 2.22E-03 |
| 112 | LMCD1 | 1.51 | Up | 3.68 | -1.27 | 2.24E-03 |
| 113 | DCLK1 | 1.68 | Up | 3.68 | -1.28 | 2.25E-03 |
| 114 | PRELP | 3.3 | Up | 3.68 | -1.28 | 2.25E-03 |
| 115 | C10orf116 | 2.58 | Up | 3.68 | -1.28 | 2.26E-03 |
| 116 | SOX10 | 1.11 | Up | 3.68 | -1.28 | 2.27E-03 |
| 117 | NKX3-2 | 1.28 | Up | 3.67 | -1.29 | 2.29E-03 |
| 118 | S100B | 1.78 | Up | 3.67 | -1.29 | 2.29E-03 |
| 119 | RELN | 1.19 | Up | 3.67 | -1.3 | 2.31E-03 |
| 120 | ETNK1 | -1.32 | Down | -3.67 | -1.3 | 2.31E-03 |
| 121 | PTPLAD1 | -1.16 | Down | -3.65 | -1.32 | 2.38E-03 |
| 122 | DUSP1 | 1.8 | Up | 3.65 | -1.34 | 2.41E-03 |
| 123 | SCG2 | 1.87 | Up | 3.64 | -1.35 | 2.44E-03 |
| 124 | NGFR | 1.41 | Up | 3.64 | -1.35 | 2.46E-03 |
| 125 | COL8A2 | 1.94 | Up | 3.63 | -1.36 | 2.49E-03 |
| 126 | FMO2 | 1.34 | Up | 3.62 | -1.38 | 2.53E-03 |
| 127 | MYOC | 1.88 | Up | 3.62 | -1.38 | 2.53E-03 |
| 128 | REEP1 | 1.68 | Up | 3.62 | -1.38 | 2.54E-03 |
| 129 | TPD52 | -2.06 | Down | -3.61 | -1.39 | 2.57E-03 |
| 130 | TPSAB1 | 1.61 | Up | 3.61 | -1.4 | 2.59E-03 |
| 131 | SGCA | 1.41 | Up | 3.61 | -1.4 | 2.61E-03 |
| 132 | PTGES | 1.08 | Up | 3.6 | -1.41 | 2.63E-03 |
| 133 | NXPH3 | 1.11 | Up | 3.59 | -1.44 | 2.71E-03 |
| 134 | HOXC10 | 1.85 | Up | 3.58 | -1.44 | 2.73E-03 |
| 135 | KIAA0485 | 1.12 | Up | 3.58 | -1.45 | 2.74E-03 |
| 136 | B4GALT4 | -1.26 | Down | -3.58 | -1.45 | 2.76E-03 |
| 137 | FLNC | 1.16 | Up | 3.57 | -1.47 | 2.80E-03 |
| 138 | SLIT2 | 1.56 | Up | 3.56 | -1.48 | 2.85E-03 |
| 139 | PCP4 | 3.23 | Up | 3.56 | -1.48 | 2.86E-03 |
| 140 | NDUFA4L2 | 1.08 | Up | 3.55 | -1.5 | 2.92E-03 |
| 141 | MBP | 1.38 | Up | 3.53 | -1.55 | 3.08E-03 |
| 142 | SYNM | 2.35 | Up | 3.52 | -1.55 | 3.09E-03 |
| 143 | GPC3 | 3.06 | Up | 3.52 | -1.56 | 3.10E-03 |
| 144 | ADCY7 | -1.06 | Down | -3.51 | -1.57 | 3.17E-03 |
| 145 | LYVE1 | 1.93 | Up | 3.51 | -1.58 | 3.18E-03 |
| 146 | MMRN2 | 1.6 | Up | 3.51 | -1.58 | 3.20E-03 |
| 147 | LYPLA1 | -1.19 | Down | -3.5 | -1.59 | 3.23E-03 |
| 148 | C7 | 4.01 | Up | 3.5 | -1.59 | 3.23E-03 |
| 149 | ZWINT | -1.88 | Down | -3.49 | -1.61 | 3.29E-03 |
| 150 | MELK | -2.06 | Down | -3.48 | -1.62 | 3.35E-03 |
| 151 | MEX3C | -1.06 | Down | -3.48 | -1.62 | 3.36E-03 |
| 152 | FZD5 | -1.46 | Down | -3.47 | -1.64 | 3.43E-03 |
| 153 | IGKV4-1 | 2.03 | Up | 3.47 | -1.66 | 3.49E-03 |
| 154 | HSPB2 | 1.31 | Up | 3.46 | -1.67 | 3.52E-03 |
| 155 | ZFP36 | 2 | Up | 3.45 | -1.69 | 3.60E-03 |
| 156 | TNS1 | 1.54 | Up | 3.45 | -1.69 | 3.63E-03 |
| 157 | SYNPO | 2.22 | Up | 3.44 | -1.69 | 3.64E-03 |
| 158 | ACTG2 | 3.63 | Up | 3.44 | -1.71 | 3.70E-03 |
| 159 | C14orf139 | 1.28 | Up | 3.43 | -1.72 | 3.73E-03 |
| 160 | HP | 2.18 | Up | 3.43 | -1.72 | 3.74E-03 |
| 161 | RAPGEF3 | 1.14 | Up | 3.43 | -1.72 | 3.74E-03 |
| 162 | HLF | 1.01 | Up | 3.43 | -1.72 | 3.76E-03 |
| 163 | CDH19 | 1.36 | Up | 3.42 | -1.75 | 3.86E-03 |
| 164 | PRPH | 1.02 | Up | 3.41 | -1.75 | 3.89E-03 |
| 165 | PNOC | 1.39 | Up | 3.41 | -1.75 | 3.89E-03 |
| 166 | DPEP2 | 1.06 | Up | 3.4 | -1.77 | 3.98E-03 |
| 167 | TPM2 | 1.29 | Up | 3.4 | -1.78 | 3.99E-03 |
| 168 | LHFP | 1.26 | Up | 3.4 | -1.78 | 3.99E-03 |
| 169 | PPP1R12B | 1.95 | Up | 3.39 | -1.79 | 4.04E-03 |
| 170 | FHL1 | 1.46 | Up | 3.38 | -1.81 | 4.17E-03 |
| 171 | ARFGAP3 | -1.21 | Down | -3.37 | -1.82 | 4.22E-03 |
| 172 | MICAL2 | 1.15 | Up | 3.37 | -1.83 | 4.24E-03 |
| 173 | NLGN4X | -1.06 | Down | -3.37 | -1.83 | 4.24E-03 |
| 174 | NGF | 1.23 | Up | 3.37 | -1.83 | 4.26E-03 |
| 175 | BMP4 | 1.03 | Up | 3.36 | -1.84 | 4.28E-03 |
| 176 | CD40 | 1.1 | Up | 3.35 | -1.86 | 4.42E-03 |
| 177 | BUB1 | -1.96 | Down | -3.35 | -1.87 | 4.45E-03 |
| 178 | EZH2 | -1.68 | Down | -3.35 | -1.87 | 4.45E-03 |
| 179 | ENPEP | -1.23 | Down | -3.34 | -1.88 | 4.49E-03 |
| 180 | SSPN | 1.03 | Up | 3.34 | -1.89 | 4.53E-03 |
| 181 | TSPAN7 | 1.38 | Up | 3.33 | -1.9 | 4.62E-03 |
| 182 | MFAP5 | 2.39 | Up | 3.32 | -1.91 | 4.66E-03 |
| 183 | PAICS | -1.2 | Down | -3.32 | -1.91 | 4.67E-03 |
| 184 | TBXA2R | 1.18 | Up | 3.32 | -1.92 | 4.70E-03 |
| 185 | ITGB1BP2 | 1.27 | Up | 3.31 | -1.94 | 4.79E-03 |
| 186 | CLEC10A | 1.3 | Up | 3.3 | -1.95 | 4.89E-03 |
| 187 | DTL | -1.83 | Down | -3.3 | -1.95 | 4.90E-03 |
| 188 | PDLIM5 | 1.6 | Up | 3.3 | -1.96 | 4.92E-03 |
| 189 | F2R | -1.38 | Down | -3.3 | -1.96 | 4.93E-03 |
| 190 | SEMA3G | 1.24 | Up | 3.29 | -1.96 | 4.95E-03 |
| 191 | NMT2 | -1 | Down | -3.29 | -1.97 | 4.99E-03 |
| 192 | KLRD1 | -1.14 | Down | -3.28 | -1.99 | 5.09E-03 |
| 193 | C3 | 1.77 | Up | 3.28 | -1.99 | 5.11E-03 |
| 194 | CFH | 1.94 | Up | 3.28 | -1.99 | 5.13E-03 |
| 195 | NR3C1 | 1.02 | Up | 3.28 | -2 | 5.14E-03 |
| 196 | ELOVL6 | -1.32 | Down | -3.27 | -2 | 5.17E-03 |
| 197 | IGLL3 | 1.52 | Up | 3.26 | -2.02 | 5.26E-03 |
| 198 | S1PR1 | 1.09 | Up | 3.26 | -2.02 | 5.29E-03 |
| 199 | GFPT1 | -1.11 | Down | -3.25 | -2.05 | 5.45E-03 |
| 200 | FBXO5 | -1.26 | Down | -3.24 | -2.07 | 5.57E-03 |
| 201 | NCAM1 | 1.07 | Up | 3.23 | -2.07 | 5.59E-03 |
| 202 | FEN1 | -1.11 | Down | -3.23 | -2.08 | 5.65E-03 |
| 203 | FCER1A | 1.26 | Up | 3.23 | -2.08 | 5.68E-03 |
| 204 | ITGA6 | -1.15 | Down | -3.22 | -2.09 | 5.71E-03 |
| 205 | AGT | 1.14 | Up | 3.22 | -2.1 | 5.76E-03 |
| 206 | AKR1C1 | 1.87 | Up | 3.22 | -2.1 | 5.76E-03 |
| 207 | ZFHX4 | 1.77 | Up | 3.22 | -2.1 | 5.78E-03 |
| 208 | SLC39A6 | -1.89 | Down | -3.22 | -2.1 | 5.81E-03 |
| 209 | RAD51 | -1.24 | Down | -3.21 | -2.12 | 5.93E-03 |
| 210 | CFP | 1.15 | Up | 3.2 | -2.12 | 5.94E-03 |
| 211 | CDC6 | -1.71 | Down | -3.2 | -2.12 | 5.95E-03 |
| 212 | LMOD1 | 1.45 | Up | 3.2 | -2.13 | 5.98E-03 |
| 213 | ACN9 | -1.01 | Down | -3.2 | -2.13 | 6.01E-03 |
| 214 | GREM1 | 2.19 | Up | 3.19 | -2.16 | 6.18E-03 |
| 215 | PRKCDBP | 1.07 | Up | 3.18 | -2.16 | 6.20E-03 |
| 216 | FBLN1 | 1.02 | Up | 3.18 | -2.16 | 6.21E-03 |
| 217 | TOP2A | -2.5 | Down | -3.18 | -2.17 | 6.25E-03 |
| 218 | RACGAP1 | -1.35 | Down | -3.18 | -2.17 | 6.27E-03 |
| 219 | EPB41L4B | -1.27 | Down | -3.18 | -2.17 | 6.28E-03 |
| 220 | NETO2 | -1.42 | Down | -3.17 | -2.18 | 6.33E-03 |
| 221 | QPCT | -1.62 | Down | -3.17 | -2.18 | 6.37E-03 |
| 222 | STYK1 | -1.26 | Down | -3.17 | -2.19 | 6.40E-03 |
| 223 | CDC25A | -1.17 | Down | -3.17 | -2.19 | 6.43E-03 |
| 224 | GGT5 | 1.29 | Up | 3.16 | -2.2 | 6.45E-03 |
| 225 | FABP4 | 2.98 | Up | 3.16 | -2.2 | 6.46E-03 |
| 226 | POPDC2 | 1.16 | Up | 3.16 | -2.2 | 6.47E-03 |
| 227 | SCD | -1.4 | Down | -3.15 | -2.22 | 6.60E-03 |
| 228 | NUSAP1 | -2.17 | Down | -3.15 | -2.22 | 6.63E-03 |
| 229 | AKR1C2 | 1.61 | Up | 3.13 | -2.25 | 6.88E-03 |
| 230 | FOS | 3.08 | Up | 3.13 | -2.25 | 6.89E-03 |
| 231 | ACTA2 | 1.58 | Up | 3.13 | -2.25 | 6.89E-03 |
| 232 | APOLD1 | 1.32 | Up | 3.13 | -2.26 | 6.91E-03 |
| 233 | NUDT21 | -1.18 | Down | -3.13 | -2.26 | 6.96E-03 |
| 234 | COL4A3 | 1.94 | Up | 3.12 | -2.27 | 7.02E-03 |
| 235 | MPZ | 1.71 | Up | 3.12 | -2.27 | 7.04E-03 |
| 236 | AMMECR1 | -1.18 | Down | -3.12 | -2.27 | 7.04E-03 |
| 237 | CMA1 | 1.4 | Up | 3.12 | -2.27 | 7.05E-03 |
| 238 | CYR61 | 1.66 | Up | 3.12 | -2.28 | 7.10E-03 |
| 239 | RRAD | 1.11 | Up | 3.11 | -2.29 | 7.18E-03 |
| 240 | RCBTB1 | -1.07 | Down | -3.11 | -2.3 | 7.24E-03 |
| 241 | CCT2 | -1.16 | Down | -3.11 | -2.3 | 7.26E-03 |
| 242 | ABI3BP | 1.2 | Up | 3.1 | -2.31 | 7.36E-03 |
| 243 | PPP2R5C | -1.04 | Down | -3.1 | -2.31 | 7.37E-03 |
| 244 | IGL | 2.55 | Up | 3.09 | -2.32 | 7.48E-03 |
| 245 | TOM1L1 | -1.32 | Down | -3.09 | -2.33 | 7.49E-03 |
| 246 | MAB21L2 | 1.23 | Up | 3.09 | -2.33 | 7.51E-03 |
| 247 | ICAM1 | 1.12 | Up | 3.09 | -2.33 | 7.54E-03 |
| 248 | CBX7 | 1.12 | Up | 3.07 | -2.35 | 7.75E-03 |
| 249 | AURKA | -1.38 | Down | -3.07 | -2.36 | 7.79E-03 |
| 250 | HSPD1 | -1.48 | Down | -3.07 | -2.36 | 7.82E-03 |
| 251 | MYOZ3 | 1.6 | Up | 3.07 | -2.37 | 7.88E-03 |
| 252 | EGR1 | 2.32 | Up | 3.06 | -2.37 | 7.91E-03 |
| 253 | TIE1 | 1.12 | Up | 3.06 | -2.37 | 7.92E-03 |
| 254 | E2F5 | -1 | Down | -3.05 | -2.4 | 8.20E-03 |
| 255 | TPX2 | -1.98 | Down | -3.04 | -2.41 | 8.25E-03 |
| 256 | TMEM47 | 1.33 | Up | 3.04 | -2.41 | 8.27E-03 |
| 257 | ABAT | -1.2 | Down | -3.04 | -2.42 | 8.31E-03 |
| 258 | ADIPOQ | 1.69 | Up | 3.04 | -2.42 | 8.33E-03 |
| 259 | SYN2 | 1.22 | Up | 3.04 | -2.42 | 8.38E-03 |
| 260 | ISL1 | 1.35 | Up | 3.04 | -2.43 | 8.40E-03 |
| 261 | RGS2 | 2.16 | Up | 3.03 | -2.43 | 8.45E-03 |
| 262 | SLC18A2 | -1.22 | Down | -3.03 | -2.43 | 8.48E-03 |
| 263 | LEPR | 1.43 | Up | 3.03 | -2.43 | 8.48E-03 |
| 264 | ATP2C1 | -1.01 | Down | -3.03 | -2.43 | 8.49E-03 |
| 265 | FCN1 | 1.68 | Up | 3.03 | -2.44 | 8.54E-03 |
| 266 | PDGFRL | 1.36 | Up | 3.02 | -2.45 | 8.67E-03 |
| 267 | RPL37A | -1.09 | Down | -3.02 | -2.46 | 8.72E-03 |
| 268 | TPSB2 | 1.91 | Up | 3.02 | -2.46 | 8.73E-03 |
| 269 | SPOCK2 | 1.17 | Up | 3.01 | -2.46 | 8.78E-03 |
| 270 | FOSB | 2.98 | Up | 3.01 | -2.47 | 8.82E-03 |
| 271 | SPAG1 | -1.15 | Down | -3.01 | -2.47 | 8.86E-03 |
| 272 | KLF2 | 2.1 | Up | 3 | -2.48 | 8.95E-03 |
| 273 | LAG3 | 1.19 | Up | 3 | -2.49 | 9.07E-03 |
| 274 | PLXDC1 | 1.24 | Up | 3 | -2.49 | 9.07E-03 |
| 275 | KCNMA1 | 1.48 | Up | 3 | -2.49 | 9.09E-03 |
| 276 | TBX3 | -1.09 | Down | -2.99 | -2.5 | 9.16E-03 |
| 277 | PDGFA | 1.08 | Up | 2.99 | -2.51 | 9.22E-03 |
| 278 | COL14A1 | 1.09 | Up | 2.99 | -2.51 | 9.26E-03 |
| 279 | FCGR2B | 1.52 | Up | 2.98 | -2.52 | 9.34E-03 |
| 280 | KIAA0101 | -2.22 | Down | -2.98 | -2.52 | 9.37E-03 |
| 281 | SHCBP1 | -1.36 | Down | -2.98 | -2.52 | 9.38E-03 |
| 282 | CCL11 | 1.18 | Up | 2.98 | -2.53 | 9.47E-03 |
| 283 | RCAN2 | 1.51 | Up | 2.96 | -2.55 | 9.73E-03 |
| 284 | MSH6 | -1.09 | Down | -2.96 | -2.56 | 9.79E-03 |
| 285 | ROD1 | -1.02 | Down | -2.96 | -2.56 | 9.85E-03 |
| 286 | YEATS4 | -1.01 | Down | -2.95 | -2.57 | 9.92E-03 |
| 287 | TENC1 | 1.03 | Up | 2.95 | -2.57 | 9.92E-03 |
| 288 | BUB1B | -2 | Down | -2.95 | -2.58 | 1.00E-02 |
| 289 | PDLIM7 | 1.05 | Up | 2.95 | -2.58 | 1.00E-02 |
| 290 | HGF | 1.05 | Up | 2.94 | -2.59 | 1.01E-02 |
| 291 | APOBEC3B | -1.23 | Down | -2.94 | -2.59 | 1.02E-02 |
| 292 | RERGL | 1.57 | Up | 2.93 | -2.62 | 1.04E-02 |
| 293 | NFIB | 1.01 | Up | 2.92 | -2.63 | 1.06E-02 |
| 294 | ACSM3 | -1.29 | Down | -2.91 | -2.64 | 1.08E-02 |
| 295 | ALOX5AP | 1.02 | Up | 2.91 | -2.65 | 1.08E-02 |
| 296 | PCOLCE2 | 1.85 | Up | 2.91 | -2.65 | 1.09E-02 |
| 297 | SLC30A5 | -1.18 | Down | -2.91 | -2.65 | 1.09E-02 |
| 298 | MAL | 1.19 | Up | 2.9 | -2.67 | 1.11E-02 |
| 299 | MCCC2 | -1 | Down | -2.9 | -2.67 | 1.11E-02 |
| 300 | TNXB | 1.3 | Up | 2.89 | -2.68 | 1.13E-02 |
| 301 | GPM6A | 1.14 | Up | 2.89 | -2.69 | 1.13E-02 |
| 302 | CLDN5 | 1.45 | Up | 2.88 | -2.69 | 1.14E-02 |
| 303 | KIF2A | -1 | Down | -2.88 | -2.69 | 1.14E-02 |
| 304 | SLIT3 | 1.29 | Up | 2.88 | -2.7 | 1.15E-02 |
| 305 | PTRF | 1.02 | Up | 2.88 | -2.71 | 1.16E-02 |
| 306 | CCNB2 | -1.9 | Down | -2.87 | -2.72 | 1.18E-02 |
| 307 | SLC25A15 | -1.01 | Down | -2.86 | -2.73 | 1.19E-02 |
| 308 | FXYD6 | 1.31 | Up | 2.86 | -2.74 | 1.20E-02 |
| 309 | KCNH2 | 1.18 | Up | 2.85 | -2.75 | 1.22E-02 |
| 310 | TAGLN | 1.84 | Up | 2.85 | -2.76 | 1.23E-02 |
| 311 | TYMS | -1.65 | Down | -2.85 | -2.76 | 1.23E-02 |
| 312 | NFASC | 2.24 | Up | 2.84 | -2.76 | 1.24E-02 |
| 313 | CTGF | 1.05 | Up | 2.84 | -2.76 | 1.24E-02 |
| 314 | FAM20B | 2.28 | Up | 2.84 | -2.77 | 1.25E-02 |
| 315 | F5 | 1.02 | Up | 2.84 | -2.77 | 1.25E-02 |
| 316 | CCRL1 | 1.01 | Up | 2.84 | -2.77 | 1.25E-02 |
| 317 | IL7R | 1.22 | Up | 2.83 | -2.78 | 1.26E-02 |
| 318 | KIF4A | -1.66 | Down | -2.83 | -2.78 | 1.26E-02 |
| 319 | C9orf82 | -1.07 | Down | -2.83 | -2.78 | 1.27E-02 |
| 320 | TOX3 | -1.29 | Down | -2.82 | -2.81 | 1.31E-02 |
| 321 | SULT1C2 | -1.08 | Down | -2.81 | -2.82 | 1.33E-02 |
| 322 | CEP55 | -1.92 | Down | -2.8 | -2.83 | 1.34E-02 |
| 323 | CRISP2 | -1.11 | Down | -2.8 | -2.83 | 1.34E-02 |
| 324 | MRPL19 | -1.01 | Down | -2.8 | -2.84 | 1.36E-02 |
| 325 | GATA6 | 2.94 | Up | 2.79 | -2.85 | 1.38E-02 |
| 326 | IGLJ3 | 1.29 | Up | 2.79 | -2.86 | 1.39E-02 |
| 327 | SGCD | 1.1 | Up | 2.78 | -2.87 | 1.40E-02 |
| 328 | CCNB1 | -1.64 | Down | -2.78 | -2.87 | 1.40E-02 |
| 329 | CES1 | 1.26 | Up | 2.78 | -2.87 | 1.41E-02 |
| 330 | LY96 | 1.05 | Up | 2.77 | -2.88 | 1.42E-02 |
| 331 | FRY | 1.11 | Up | 2.77 | -2.88 | 1.42E-02 |
| 332 | IGHM | 2 | Up | 2.77 | -2.89 | 1.44E-02 |
| 333 | TRIP13 | -1.22 | Down | -2.77 | -2.89 | 1.44E-02 |
| 334 | SORD | -1.51 | Down | -2.76 | -2.9 | 1.45E-02 |
| 335 | GINS1 | -1.51 | Down | -2.76 | -2.9 | 1.45E-02 |
| 336 | SLC11A2 | -1.19 | Down | -2.76 | -2.91 | 1.46E-02 |
| 337 | CPB1 | 1.01 | Up | 2.76 | -2.91 | 1.47E-02 |
| 338 | UPK3B | 1.41 | Up | 2.76 | -2.91 | 1.47E-02 |
| 339 | FABP3 | 1.54 | Up | 2.75 | -2.92 | 1.48E-02 |
| 340 | MCM4 | -1.06 | Down | -2.75 | -2.93 | 1.50E-02 |
| 341 | BCHE | 2 | Up | 2.74 | -2.94 | 1.52E-02 |
| 342 | RARRES1 | 1.25 | Up | 2.74 | -2.95 | 1.53E-02 |
| 343 | C14orf109 | -1.01 | Down | -2.74 | -2.95 | 1.53E-02 |
| 344 | IVNS1ABP | -1.18 | Down | -2.74 | -2.95 | 1.54E-02 |
| 345 | KIF20A | -2.07 | Down | -2.73 | -2.96 | 1.55E-02 |
| 346 | LTC4S | 1.28 | Up | 2.73 | -2.96 | 1.56E-02 |
| 347 | MXRA5 | 1.3 | Up | 2.73 | -2.96 | 1.56E-02 |
| 348 | TMEM48 | -1.1 | Down | -2.73 | -2.97 | 1.57E-02 |
| 349 | SLC16A1 | -1.16 | Down | -2.72 | -2.97 | 1.57E-02 |
| 350 | ATMIN | -1.06 | Down | -2.72 | -2.97 | 1.58E-02 |
| 351 | CCL23 | 1.57 | Up | 2.72 | -2.98 | 1.59E-02 |
| 352 | SOD3 | 1.45 | Up | 2.72 | -2.98 | 1.59E-02 |
| 353 | MYL9 | 1.54 | Up | 2.71 | -2.99 | 1.61E-02 |
| 354 | KIF11 | -1.78 | Down | -2.71 | -2.99 | 1.61E-02 |
| 355 | CENPA | -1.91 | Down | -2.71 | -2.99 | 1.61E-02 |
| 356 | IGFBP5 | 1.04 | Up | 2.71 | -2.99 | 1.61E-02 |
| 357 | CDK1 | -1.76 | Down | -2.71 | -3 | 1.62E-02 |
| 358 | GTSE1 | -1.64 | Down | -2.71 | -3 | 1.63E-02 |
| 359 | PLA2G7 | -1.51 | Down | -2.71 | -3 | 1.63E-02 |
| 360 | BGN | 1.35 | Up | 2.71 | -3 | 1.63E-02 |
| 361 | TAF7L | -1.07 | Down | -2.7 | -3 | 1.64E-02 |
| 362 | POLE2 | -1.26 | Down | -2.7 | -3.01 | 1.64E-02 |
| 363 | FAM107A | 1.66 | Up | 2.7 | -3.02 | 1.66E-02 |
| 364 | CENPN | -1.52 | Down | -2.7 | -3.02 | 1.67E-02 |
| 365 | CTSG | 1.68 | Up | 2.69 | -3.03 | 1.68E-02 |
| 366 | CDC7 | -1.2 | Down | -2.69 | -3.03 | 1.68E-02 |
| 367 | DSG2 | -1.06 | Down | -2.69 | -3.04 | 1.70E-02 |
| 368 | PLS1 | -1.15 | Down | -2.68 | -3.04 | 1.71E-02 |
| 369 | CRYAB | 1.25 | Up | 2.67 | -3.06 | 1.75E-02 |
| 370 | PRC1 | -1.5 | Down | -2.67 | -3.06 | 1.75E-02 |
| 371 | PECAM1 | 1.09 | Up | 2.67 | -3.07 | 1.76E-02 |
| 372 | CKS2 | -1.45 | Down | -2.67 | -3.07 | 1.77E-02 |
| 373 | MFAP4 | 1.43 | Up | 2.66 | -3.08 | 1.79E-02 |
| 374 | MYLK | 1.26 | Up | 2.65 | -3.1 | 1.82E-02 |
| 375 | DNAJC9 | -1.02 | Down | -2.65 | -3.1 | 1.82E-02 |
| 376 | ACTC1 | 1.7 | Up | 2.65 | -3.11 | 1.84E-02 |
| 377 | TSC22D3 | 1.29 | Up | 2.64 | -3.11 | 1.85E-02 |
| 378 | PLCH1 | -1.21 | Down | -2.63 | -3.13 | 1.90E-02 |
| 379 | AEBP1 | 1.3 | Up | 2.63 | -3.14 | 1.90E-02 |
| 380 | BCAP29 | -1.02 | Down | -2.63 | -3.14 | 1.91E-02 |
| 381 | GSTM5 | 1.38 | Up | 2.63 | -3.14 | 1.92E-02 |
| 382 | PCNA | -1.14 | Down | -2.61 | -3.16 | 1.97E-02 |
| 383 | PUS7 | -1.08 | Down | -2.61 | -3.16 | 1.97E-02 |
| 384 | OIP5 | -1.53 | Down | -2.61 | -3.16 | 1.97E-02 |
| 385 | MEIS2 | 1.08 | Up | 2.61 | -3.17 | 1.99E-02 |
| 386 | DPYSL3 | 1.47 | Up | 2.61 | -3.17 | 1.99E-02 |
| 387 | PLSCR4 | 1.31 | Up | 2.61 | -3.17 | 1.99E-02 |
| 388 | GIMAP5 | 1.14 | Up | 2.61 | -3.18 | 2.00E-02 |
| 389 | SLC7A1 | -1.16 | Down | -2.6 | -3.18 | 2.00E-02 |
| 390 | PGRMC1 | -1.03 | Down | -2.6 | -3.18 | 2.02E-02 |
| 391 | TSPAN12 | -1.16 | Down | -2.6 | -3.19 | 2.02E-02 |
| 392 | HMGB3 | -1.08 | Down | -2.6 | -3.19 | 2.03E-02 |
| 393 | JUNB | 1.37 | Up | 2.6 | -3.19 | 2.03E-02 |
| 394 | RAB1A | -1.09 | Down | -2.59 | -3.19 | 2.04E-02 |
| 395 | E2F8 | -1.42 | Down | -2.59 | -3.2 | 2.06E-02 |
| 396 | EIF4E | -1.03 | Down | -2.59 | -3.21 | 2.07E-02 |
| 397 | KCNB1 | 1.02 | Up | 2.58 | -3.21 | 2.08E-02 |
| 398 | RGS5 | 1.46 | Up | 2.58 | -3.21 | 2.09E-02 |
| 399 | HK2 | -1.04 | Down | -2.57 | -3.24 | 2.14E-02 |
| 400 | S100A4 | 1.07 | Up | 2.56 | -3.25 | 2.18E-02 |
| 401 | PLIN1 | 1.11 | Up | 2.56 | -3.25 | 2.18E-02 |
| 402 | SPA17 | -1.08 | Down | -2.56 | -3.26 | 2.20E-02 |
| 403 | MCM10 | -1.38 | Down | -2.55 | -3.26 | 2.21E-02 |
| 404 | EMCN | 1.11 | Up | 2.55 | -3.27 | 2.21E-02 |
| 405 | ZBTB10 | -1.11 | Down | -2.55 | -3.27 | 2.22E-02 |
| 406 | NTRK2 | 2.19 | Up | 2.55 | -3.27 | 2.22E-02 |
| 407 | LGR4 | -1.03 | Down | -2.55 | -3.27 | 2.22E-02 |
| 408 | JUN | 1.03 | Up | 2.55 | -3.27 | 2.23E-02 |
| 409 | MCAM | 1.06 | Up | 2.54 | -3.29 | 2.27E-02 |
| 410 | HLX | 1.13 | Up | 2.54 | -3.29 | 2.28E-02 |
| 411 | KCNE4 | 1.31 | Up | 2.54 | -3.29 | 2.28E-02 |
| 412 | MTR | -1.02 | Down | -2.54 | -3.29 | 2.29E-02 |
| 413 | VSIG4 | 1.24 | Up | 2.53 | -3.3 | 2.30E-02 |
| 414 | WNT2B | 1.59 | Up | 2.53 | -3.31 | 2.33E-02 |
| 415 | TIMP4 | 1.53 | Up | 2.52 | -3.32 | 2.36E-02 |
| 416 | HSD17B6 | 2.13 | Up | 2.52 | -3.33 | 2.39E-02 |
| 417 | C6orf211 | -1.11 | Down | -2.51 | -3.33 | 2.39E-02 |
| 418 | FRMD4B | -1.05 | Down | -2.51 | -3.33 | 2.39E-02 |
| 419 | CDA | 1.01 | Up | 2.51 | -3.33 | 2.39E-02 |
| 420 | CCL21 | 1.32 | Up | 2.51 | -3.34 | 2.41E-02 |
| 421 | PLA2G2A | 2.76 | Up | 2.51 | -3.34 | 2.41E-02 |
| 422 | SLC15A2 | -1.64 | Down | -2.5 | -3.36 | 2.46E-02 |
| 423 | AOX1 | 2.25 | Up | 2.5 | -3.36 | 2.47E-02 |
| 424 | CCNA2 | -1.57 | Down | -2.5 | -3.36 | 2.47E-02 |
| 425 | COL4A6 | 1.55 | Up | 2.5 | -3.36 | 2.48E-02 |
| 426 | CWH43 | -1 | Down | -2.49 | -3.37 | 2.51E-02 |
| 427 | GEM | 1.3 | Up | 2.48 | -3.38 | 2.53E-02 |
| 428 | PBK | -1.97 | Down | -2.48 | -3.38 | 2.54E-02 |
| 429 | TMEM97 | -1.25 | Down | -2.47 | -3.4 | 2.59E-02 |
| 430 | ITGBL1 | 1.12 | Up | 2.47 | -3.41 | 2.63E-02 |
| 431 | DPY19L1 | -1.03 | Down | -2.47 | -3.41 | 2.63E-02 |
| 432 | CHL1 | 1.24 | Up | 2.46 | -3.42 | 2.66E-02 |
| 433 | KIF2C | -1.23 | Down | -2.46 | -3.43 | 2.67E-02 |
| 434 | CKAP2 | -1.21 | Down | -2.46 | -3.43 | 2.68E-02 |
| 435 | ZWILCH | -1.04 | Down | -2.45 | -3.43 | 2.69E-02 |
| 436 | COL13A1 | 1.22 | Up | 2.45 | -3.44 | 2.71E-02 |
| 437 | ECT2 | -1.45 | Down | -2.44 | -3.45 | 2.75E-02 |
| 438 | SOX18 | 1.14 | Up | 2.43 | -3.47 | 2.82E-02 |
| 439 | FGF7 | 1.22 | Up | 2.43 | -3.47 | 2.82E-02 |
| 440 | MAD2L1 | -1.51 | Down | -2.43 | -3.48 | 2.83E-02 |
| 441 | GAS1 | 1.55 | Up | 2.43 | -3.48 | 2.83E-02 |
| 442 | HSD11B1 | 1.35 | Up | 2.43 | -3.48 | 2.84E-02 |
| 443 | AURKB | -1.29 | Down | -2.43 | -3.48 | 2.84E-02 |
| 444 | COL6A2 | 1.19 | Up | 2.42 | -3.48 | 2.86E-02 |
| 445 | FOXA2 | -1.14 | Down | -2.42 | -3.48 | 2.86E-02 |
| 446 | RAMP3 | 1.22 | Up | 2.42 | -3.49 | 2.88E-02 |
| 447 | ANK3 | -1.28 | Down | -2.41 | -3.5 | 2.91E-02 |
| 448 | OPN3 | -1.11 | Down | -2.41 | -3.51 | 2.94E-02 |
| 449 | ADAMTS3 | 1.08 | Up | 2.41 | -3.51 | 2.94E-02 |
| 450 | TCF21 | 2.59 | Up | 2.41 | -3.51 | 2.96E-02 |
| 451 | CHI3L1 | 1.54 | Up | 2.4 | -3.52 | 2.98E-02 |
| 452 | SEC24D | -1.02 | Down | -2.39 | -3.53 | 3.03E-02 |
| 453 | NCAPG | -1.27 | Down | -2.39 | -3.55 | 3.07E-02 |
| 454 | NAT1 | -1.05 | Down | -2.37 | -3.56 | 3.14E-02 |
| 455 | HPD | 1.09 | Up | 2.37 | -3.57 | 3.16E-02 |
| 456 | ZFPM2 | 1.39 | Up | 2.37 | -3.57 | 3.17E-02 |
| 457 | HJURP | -1.2 | Down | -2.36 | -3.58 | 3.21E-02 |
| 458 | SLMO2 | -1.14 | Down | -2.36 | -3.59 | 3.23E-02 |
| 459 | BIRC5 | -1.41 | Down | -2.35 | -3.6 | 3.27E-02 |
| 460 | PLP1 | 1.74 | Up | 2.35 | -3.6 | 3.27E-02 |
| 461 | IGKC | 1.99 | Up | 2.34 | -3.62 | 3.36E-02 |
| 462 | LRRC1 | -1.1 | Down | -2.33 | -3.64 | 3.42E-02 |
| 463 | RAD51AP1 | -1.27 | Down | -2.33 | -3.64 | 3.42E-02 |
| 464 | CACNA1G | -1.23 | Down | -2.33 | -3.64 | 3.42E-02 |
| 465 | KLRC3 | -1.09 | Down | -2.32 | -3.65 | 3.46E-02 |
| 466 | MKI67 | -1.13 | Down | -2.32 | -3.66 | 3.51E-02 |
| 467 | DBF4 | -1.17 | Down | -2.32 | -3.66 | 3.51E-02 |
| 468 | GMNN | -1.04 | Down | -2.31 | -3.67 | 3.54E-02 |
| 469 | PRLR | -1.01 | Down | -2.31 | -3.67 | 3.55E-02 |
| 470 | PTTG1 | -1.27 | Down | -2.3 | -3.69 | 3.63E-02 |
| 471 | ATAD2 | -1.07 | Down | -2.3 | -3.69 | 3.65E-02 |
| 472 | GRHL2 | -1.28 | Down | -2.29 | -3.7 | 3.67E-02 |
| 473 | PRKCQ | -1.03 | Down | -2.29 | -3.71 | 3.73E-02 |
| 474 | HOXC6 | 2.01 | Up | 2.28 | -3.71 | 3.74E-02 |
| 475 | SPAG5 | -1.07 | Down | -2.28 | -3.72 | 3.77E-02 |
| 476 | FOXM1 | -1.21 | Down | -2.28 | -3.72 | 3.77E-02 |
| 477 | PIP5K1B | -1.58 | Down | -2.27 | -3.73 | 3.83E-02 |
| 478 | SPINK2 | -1.08 | Down | -2.27 | -3.73 | 3.83E-02 |
| 479 | ASPN | 2.06 | Up | 2.26 | -3.75 | 3.90E-02 |
| 480 | IGFBP6 | 1.27 | Up | 2.26 | -3.75 | 3.90E-02 |
| 481 | NEK2 | -1.3 | Down | -2.26 | -3.75 | 3.91E-02 |
| 482 | GCLC | -1.08 | Down | -2.26 | -3.75 | 3.92E-02 |
| 483 | SH3YL1 | -1.05 | Down | -2.25 | -3.76 | 3.98E-02 |
| 484 | SPC25 | -1.41 | Down | -2.25 | -3.77 | 3.98E-02 |
| 485 | PLA2G5 | 1.86 | Up | 2.25 | -3.77 | 4.00E-02 |
| 486 | HMGCS1 | -1.04 | Down | -2.25 | -3.77 | 4.01E-02 |
| 487 | STAB2 | 1 | Up | 2.25 | -3.77 | 4.01E-02 |
| 488 | COMP | 1.99 | Up | 2.23 | -3.8 | 4.13E-02 |
| 489 | F2RL1 | -1.46 | Down | -2.23 | -3.8 | 4.15E-02 |
| 490 | TMEFF1 | -1.06 | Down | -2.23 | -3.8 | 4.17E-02 |
| 491 | BNC2 | 1.21 | Up | 2.23 | -3.81 | 4.18E-02 |
| 492 | CDC45L | -1.06 | Down | -2.23 | -3.81 | 4.18E-02 |
| 493 | WISP2 | 2.18 | Up | 2.23 | -3.81 | 4.19E-02 |
| 494 | CYP2J2 | -1.1 | Down | -2.21 | -3.83 | 4.31E-02 |
| 495 | TRIL | 1.06 | Up | 2.21 | -3.83 | 4.32E-02 |
| 496 | MLF1IP | -1.73 | Down | -2.2 | -3.85 | 4.38E-02 |
| 497 | DLGAP5 | -1.8 | Down | -2.2 | -3.85 | 4.40E-02 |
| 498 | APOD | 1.24 | Up | 2.2 | -3.85 | 4.41E-02 |
| 499 | COL10A1 | 1.2 | Up | 2.2 | -3.85 | 4.42E-02 |
| 500 | PER1 | 1.05 | Up | 2.19 | -3.86 | 4.44E-02 |
| 501 | SERPINA3 | 1.44 | Up | 2.19 | -3.86 | 4.48E-02 |
| 502 | SYNCRIP | -1.02 | Down | -2.19 | -3.87 | 4.51E-02 |
| 503 | UBE2C | -1.29 | Down | -2.19 | -3.87 | 4.51E-02 |
| 504 | CXCL2 | 1.44 | Up | 2.18 | -3.89 | 4.59E-02 |
| 505 | IDI1 | -1.05 | Down | -2.17 | -3.9 | 4.69E-02 |
| 506 | AHNAK2 | 1.42 | Up | 2.16 | -3.91 | 4.72E-02 |
| 507 | MAP7 | -1.18 | Down | -2.16 | -3.91 | 4.74E-02 |
| 508 | NPR1 | 1.06 | Up | 2.16 | -3.92 | 4.79E-02 |
| 509 | PCSK5 | -1.09 | Down | -2.15 | -3.93 | 4.82E-02 |
| 510 | CPM | -1.87 | Down | -2.15 | -3.93 | 4.83E-02 |
| 511 | ABCA8 | 1.24 | Up | 2.15 | -3.93 | 4.84E-02 |
| 512 | MPZL2 | -1.44 | Down | -2.15 | -3.93 | 4.85E-02 |
| 513 | PRG4 | 1.39 | Up | 2.15 | -3.93 | 4.87E-02 |
| 514 | RNASE1 | 1.12 | Up | 2.14 | -3.94 | 4.89E-02 |
| 515 | ADAM12 | -1.48 | Down | -2.14 | -3.94 | 4.90E-02 |
| 516 | FDX1 | -1.01 | Down | -2.13 | -3.95 | 4.99E-02 |

NA, not available.
