# Supplementary material for: Comprehensive characterization of endometrial competing endogenous RNA network in infertile women of childbearing age
Source: Aging (Albany NY). 2020 Feb 29;12(5):4204–21. doi: 10.18632/aging.102874 (PMC7093184; doi:10.18632/aging.102874)
Supplement: Supplementary Table 2 [file aging-12-102874-s001..docx]

**Supplementary Table 2. Full list of differentially expressed miRNAs in Ectopic Endometrium.**

| **ID** | **Gene_Symbol** | **log_2_(Fold Change)** | **Regulation** | **t Value** | **B Value** | **P Value** |
| --- | --- | --- | --- | --- | --- | --- |
| 1 | miR-141-5p | 5.13 | Up | 13.37 | 11.81 | 2.51E-09 |
| 2 | miR-200c-3p | 4.64 | Up | 13.07 | 11.53 | 3.38E-09 |
| 3 | miR-708-5p | -3.51 | Down | -12.87 | 11.34 | 4.12E-09 |
| 4 | miR-34c-3p | 4.78 | Up | 12.76 | 11.24 | 4.60E-09 |
| 5 | miR-449b-5p | 6.80 | Up | 12.68 | 11.17 | 4.97E-09 |
| 6 | miR-200a-3p | 5.05 | Up | 12.08 | 10.57 | 9.23E-09 |
| 7 | miR-34b-5p | 4.85 | Up | 11.78 | 10.26 | 1.28E-08 |
| 8 | miR-141-3p | 5.62 | Up | 11.70 | 10.18 | 1.39E-08 |
| 9 | miR-200a-5p | 4.79 | Up | 10.89 | 9.29 | 3.46E-08 |
| 10 | miR-200b-5p | 4.48 | Up | 10.84 | 9.24 | 3.67E-08 |
| 11 | miR-216a-5p | -4.94 | Down | -10.56 | 8.92 | 5.06E-08 |
| 12 | miR-223-3p | -3.38 | Down | -10.51 | 8.86 | 5.37E-08 |
| 13 | miR-449c-5p | 6.69 | Up | 10.27 | 8.58 | 7.19E-08 |
| 14 | miR-29c-3p | -1.80 | Down | -10.24 | 8.55 | 7.40E-08 |
| 15 | miR-202-5p | -8.27 | Down | -9.98 | 8.24 | 1.01E-07 |
| 16 | miR-10a-5p | 3.85 | Up | 9.68 | 7.86 | 1.48E-07 |
| 17 | miR-449a | 6.41 | Up | 9.58 | 7.74 | 1.67E-07 |
| 18 | miR-34c-5p | 4.30 | Up | 9.46 | 7.58 | 1.96E-07 |
| 19 | miR-767-5p | 6.30 | Up | 10.53 | 7.58 | 2.19E-07 |
| 20 | miR-196b-5p | 3.89 | Up | 9.22 | 7.28 | 2.67E-07 |
| 21 | miR-375 | 7.05 | Up | 9.08 | 7.10 | 3.20E-07 |
| 22 | miR-214-3p | -1.77 | Down | -9.07 | 7.08 | 3.26E-07 |
| 23 | miR-196b-3p | 4.23 | Up | 8.99 | 6.97 | 3.62E-07 |
| 24 | miR-675-3p | 3.54 | Up | 8.85 | 6.78 | 4.38E-07 |
| 25 | miR-10a-3p | 3.61 | Up | 8.76 | 6.66 | 4.97E-07 |
| 26 | miR-592 | -3.14 | Down | -8.67 | 6.54 | 5.59E-07 |
| 27 | miR-217 | -4.78 | Down | -8.64 | 6.50 | 5.80E-07 |
| 28 | miR-133a-3p | -2.47 | Down | -8.55 | 6.38 | 6.57E-07 |
| 29 | miR-708-3p | -2.64 | Down | -8.46 | 6.26 | 7.43E-07 |
| 30 | miR-200b-3p | 3.96 | Up | 8.34 | 6.08 | 8.89E-07 |
| 31 | miR-106a-5p | 2.37 | Up | 8.09 | 5.73 | 1.26E-06 |
| 32 | miR-203a-3p | 3.94 | Up | 7.98 | 5.57 | 1.47E-06 |
| 33 | miR-3664-3p | 3.69 | Up | 8.69 | 5.58 | 1.69E-06 |
| 34 | miR-561-5p | 1.73 | Up | 7.88 | 5.42 | 1.72E-06 |
| 35 | miR-34b-3p | 4.03 | Up | 7.85 | 5.37 | 1.80E-06 |
| 36 | miR-615-3p | -4.76 | Down | -7.68 | 5.12 | 2.29E-06 |
| 37 | miR-200c-5p | 4.70 | Up | 8.93 | 5.30 | 2.41E-06 |
| 38 | miR-6500-3p | 1.84 | Up | 7.54 | 4.91 | 2.84E-06 |
| 39 | miR-1295a | -3.45 | Down | -7.97 | 4.70 | 4.10E-06 |
| 40 | miR-145-3p | -1.75 | Down | -7.16 | 4.32 | 5.09E-06 |
| 41 | miR-485-3p | -1.56 | Down | -7.04 | 4.15 | 6.05E-06 |
| 42 | miR-539-3p | -1.56 | Down | -6.85 | 3.84 | 8.16E-06 |
| 43 | miR-133b | -3.25 | Down | -6.77 | 3.71 | 9.32E-06 |
| 44 | miR-143-5p | -1.58 | Down | -6.72 | 3.63 | 1.01E-05 |
| 45 | miR-203b-3p | 4.00 | Up | 7.60 | 3.79 | 1.11E-05 |
| 46 | miR-182-5p | 3.00 | Up | 6.57 | 3.38 | 1.29E-05 |
| 47 | miR-493-5p | -1.75 | Down | -6.52 | 3.29 | 1.41E-05 |
| 48 | miR-134-3p | -2.16 | Down | -6.47 | 3.22 | 1.51E-05 |
| 49 | miR-190b | 2.59 | Up | 6.47 | 3.21 | 1.52E-05 |
| 50 | miR-382-5p | -1.51 | Down | -6.47 | 3.21 | 1.53E-05 |
| 51 | miR-18a-3p | 1.40 | Up | 6.46 | 3.20 | 1.55E-05 |
| 52 | miR-223-5p | -2.32 | Down | -6.34 | 3.00 | 1.88E-05 |
| 53 | miR-675-5p | 2.65 | Up | 6.82 | 3.14 | 1.93E-05 |
| 54 | miR-29c-5p | -1.25 | Down | -6.29 | 2.92 | 2.04E-05 |
| 55 | miR-3158-3p | 1.18 | Up | 6.29 | 2.91 | 2.05E-05 |
| 56 | miR-4645-3p | -1.52 | Down | -6.26 | 2.85 | 2.17E-05 |
| 57 | miR-182-3p | 5.02 | Up | 6.71 | 2.99 | 2.25E-05 |
| 58 | miR-154-5p | -1.60 | Down | -6.20 | 2.76 | 2.38E-05 |
| 59 | miR-874-5p | -2.22 | Down | -6.19 | 2.74 | 2.42E-05 |
| 60 | miR-183-5p | 2.87 | Up | 6.15 | 2.68 | 2.58E-05 |
| 61 | miR-30a-5p | 1.26 | Up | 6.12 | 2.62 | 2.72E-05 |
| 62 | let-7a-2-3p | -2.48 | Down | -6.10 | 2.58 | 2.83E-05 |
| 63 | miR-214-5p | -1.18 | Down | -6.04 | 2.48 | 3.14E-05 |
| 64 | miR-873-5p | 4.27 | Up | 6.02 | 2.45 | 3.23E-05 |
| 65 | miR-573 | 3.66 | Up | 6.20 | 2.50 | 3.35E-05 |
| 66 | miR-184 | 2.51 | Up | 6.19 | 2.48 | 3.39E-05 |
| 67 | miR-874-3p | -2.12 | Down | -5.94 | 2.30 | 3.74E-05 |
| 68 | miR-3616-5p | 4.19 | Up | 5.93 | 2.30 | 3.76E-05 |
| 69 | miR-345-5p | 1.80 | Up | 5.90 | 2.23 | 4.00E-05 |
| 70 | miR-20b-5p | 2.71 | Up | 5.88 | 2.20 | 4.13E-05 |
| 71 | miR-134-5p | -1.69 | Down | -5.85 | 2.15 | 4.32E-05 |
| 72 | miR-664a-3p | -1.66 | Down | -5.84 | 2.13 | 4.44E-05 |
| 73 | miR-100-5p | -1.73 | Down | -5.81 | 2.08 | 4.65E-05 |
| 74 | miR-542-5p | 1.96 | Up | 5.75 | 1.98 | 5.14E-05 |
| 75 | miR-187-3p | 3.42 | Up | 5.74 | 1.95 | 5.30E-05 |
| 76 | miR-30d-3p | 1.53 | Up | 5.67 | 1.84 | 5.91E-05 |
| 77 | miR-25-5p | 1.06 | Up | 5.64 | 1.79 | 6.22E-05 |
| 78 | miR-33b-3p | 2.08 | Up | 5.62 | 1.74 | 6.52E-05 |
| 79 | miR-425-5p | 1.04 | Up | 5.57 | 1.65 | 7.12E-05 |
| 80 | miR-514a-3p | -5.42 | Down | -5.47 | 1.47 | 8.48E-05 |
| 81 | miR-483-3p | 2.73 | Up | 5.42 | 1.37 | 9.33E-05 |
| 82 | miR-574-3p | -1.62 | Down | -5.40 | 1.34 | 9.64E-05 |
| 83 | miR-556-5p | 2.38 | Up | 5.55 | 1.42 | 9.66E-05 |
| 84 | miR-22-3p | -1.43 | Down | -5.34 | 1.25 | 1.06E-04 |
| 85 | miR-4662a-5p | -2.02 | Down | -5.34 | 1.23 | 1.08E-04 |
| 86 | miR-539-5p | -1.72 | Down | -5.33 | 1.21 | 1.10E-04 |
| 87 | miR-656-3p | -1.44 | Down | -5.31 | 1.18 | 1.13E-04 |
| 88 | miR-216a-3p | -3.48 | Down | -5.40 | 1.16 | 1.25E-04 |
| 89 | miR-17-5p | 1.15 | Up | 5.23 | 1.05 | 1.29E-04 |
| 90 | miR-338-5p | -1.75 | Down | -5.18 | 0.94 | 1.43E-04 |
| 91 | miR-203a-5p | 2.34 | Up | 5.90 | 1.25 | 1.56E-04 |
| 92 | miR-1185-5p | -1.29 | Down | -5.12 | 0.83 | 1.60E-04 |
| 93 | miR-876-5p | 5.21 | Up | 5.04 | 0.69 | 1.83E-04 |
| 94 | miR-6502-5p | -4.92 | Down | -5.32 | 0.85 | 1.86E-04 |
| 95 | miR-224-5p | 1.88 | Up | 5.01 | 0.62 | 1.96E-04 |
| 96 | miR-197-3p | -1.32 | Down | -4.98 | 0.57 | 2.08E-04 |
| 97 | miR-127-3p | -1.73 | Down | -4.97 | 0.56 | 2.09E-04 |
| 98 | miR-34a-5p | -1.26 | Down | -4.96 | 0.53 | 2.14E-04 |
| 99 | miR-628-3p | -1.17 | Down | -4.96 | 0.53 | 2.15E-04 |
| 100 | miR-455-3p | 1.30 | Up | 4.93 | 0.48 | 2.26E-04 |
| 101 | miR-1243 | -2.49 | Down | -5.03 | 0.52 | 2.35E-04 |
| 102 | miR-363-3p | 1.98 | Up | 4.91 | 0.43 | 2.36E-04 |
| 103 | miR-4524a-5p | -2.01 | Down | -4.84 | 0.31 | 2.66E-04 |
| 104 | miR-3934-5p | 1.39 | Up | 4.77 | 0.17 | 3.07E-04 |
| 105 | miR-328-3p | -1.32 | Down | -4.76 | 0.15 | 3.12E-04 |
| 106 | miR-369-3p | -1.35 | Down | -4.75 | 0.14 | 3.15E-04 |
| 107 | miR-130b-5p | 1.09 | Up | 4.72 | 0.09 | 3.33E-04 |
| 108 | miR-183-3p | 4.35 | Up | 4.68 | 0.01 | 3.58E-04 |
| 109 | miR-127-5p | -1.40 | Down | -4.66 | -0.03 | 3.74E-04 |
| 110 | miR-30d-5p | 1.30 | Up | 4.63 | -0.08 | 3.95E-04 |
| 111 | miR-193b-3p | -1.22 | Down | -4.60 | -0.15 | 4.24E-04 |
| 112 | miR-483-5p | 2.35 | Up | 4.59 | -0.17 | 4.31E-04 |
| 113 | miR-29b-2-5p | -1.21 | Down | -4.58 | -0.19 | 4.37E-04 |
| 114 | miR-381-3p | -1.37 | Down | -4.56 | -0.23 | 4.56E-04 |
| 115 | miR-22-5p | -1.54 | Down | -4.47 | -0.40 | 5.41E-04 |
| 116 | miR-143-3p | -1.26 | Down | -4.42 | -0.50 | 5.95E-04 |
| 117 | miR-655-3p | -1.35 | Down | -4.42 | -0.50 | 5.97E-04 |
| 118 | miR-199a-5p | -1.10 | Down | -4.41 | -0.50 | 5.97E-04 |
| 119 | miR-29b-3p | -1.20 | Down | -4.41 | -0.50 | 6.00E-04 |
| 120 | miR-9-5p | 1.24 | Up | 4.38 | -0.56 | 6.33E-04 |
| 121 | miR-887-3p | -1.02 | Down | -4.37 | -0.59 | 6.55E-04 |
| 122 | miR-136-3p | -1.26 | Down | -4.33 | -0.66 | 7.00E-04 |
| 123 | miR-7974 | 3.64 | Up | 4.31 | -0.70 | 7.27E-04 |
| 124 | miR-4772-3p | -2.52 | Down | -4.29 | -0.74 | 7.56E-04 |
| 125 | miR-4662a-3p | -2.77 | Down | -4.28 | -0.76 | 7.71E-04 |
| 126 | miR-509-3p | -4.05 | Down | -4.28 | -0.77 | 7.81E-04 |
| 127 | miR-192-3p | 2.99 | Up | 4.31 | -0.80 | 8.67E-04 |
| 128 | miR-202-3p | -4.38 | Down | -4.63 | -0.57 | 9.55E-04 |
| 129 | miR-106a-3p | 2.16 | Up | 4.11 | -1.08 | 1.07E-03 |
| 130 | miR-4711-5p | -2.87 | Down | -4.18 | -1.03 | 1.09E-03 |
| 131 | miR-371b-3p | -2.07 | Down | -4.08 | -1.15 | 1.14E-03 |
| 132 | miR-4735-5p | -2.56 | Down | -4.14 | -1.12 | 1.19E-03 |
| 133 | miR-487a-5p | -1.79 | Down | -4.05 | -1.22 | 1.22E-03 |
| 134 | miR-138-5p | -2.70 | Down | -4.04 | -1.22 | 1.22E-03 |
| 135 | miR-2355-5p | -1.39 | Down | -3.99 | -1.32 | 1.35E-03 |
| 136 | miR-503-5p | 1.63 | Up | 3.99 | -1.32 | 1.36E-03 |
| 137 | miR-889-3p | -1.16 | Down | -3.98 | -1.35 | 1.39E-03 |
| 138 | miR-3591-5p | -4.02 | Down | -3.96 | -1.38 | 1.43E-03 |
| 139 | miR-653-3p | -2.54 | Down | -4.01 | -1.35 | 1.50E-03 |
| 140 | miR-146b-3p | -1.22 | Down | -3.94 | -1.42 | 1.50E-03 |
| 141 | miR-216b-5p | -3.90 | Down | -3.94 | -1.43 | 1.50E-03 |
| 142 | miR-556-3p | 1.77 | Up | 4.08 | -1.30 | 1.55E-03 |
| 143 | miR-934 | 2.72 | Up | 4.17 | -1.21 | 1.58E-03 |
| 144 | miR-3921 | -3.52 | Down | -3.97 | -1.43 | 1.63E-03 |
| 145 | miR-3126-3p | -1.58 | Down | -3.89 | -1.51 | 1.64E-03 |
| 146 | miR-27a-5p | 1.80 | Up | 3.84 | -1.61 | 1.81E-03 |
| 147 | miR-21-3p | -1.03 | Down | -3.84 | -1.62 | 1.83E-03 |
| 148 | miR-3176 | 1.77 | Up | 3.82 | -1.65 | 1.88E-03 |
| 149 | miR-4792 | -1.44 | Down | -3.79 | -1.71 | 2.01E-03 |
| 150 | miR-18b-5p | 1.55 | Up | 3.79 | -1.72 | 2.01E-03 |
| 151 | miR-9-3p | 1.05 | Up | 3.77 | -1.76 | 2.11E-03 |
| 152 | miR-3129-3p | -1.48 | Down | -3.83 | -1.70 | 2.12E-03 |
| 153 | miR-1266-5p | 3.15 | Up | 3.82 | -1.71 | 2.15E-03 |
| 154 | miR-491-3p | 2.24 | Up | 3.75 | -1.79 | 2.16E-03 |
| 155 | miR-92a-1-5p | 1.29 | Up | 3.73 | -1.83 | 2.25E-03 |
| 156 | miR-301b-3p | 1.09 | Up | 3.72 | -1.85 | 2.30E-03 |
| 157 | miR-513c-5p | -3.60 | Down | -3.76 | -1.83 | 2.43E-03 |
| 158 | miR-191-3p | 1.29 | Up | 3.67 | -1.95 | 2.53E-03 |
| 159 | miR-100-3p | -1.22 | Down | -3.66 | -1.96 | 2.58E-03 |
| 160 | miR-542-3p | 1.17 | Up | 3.63 | -2.03 | 2.77E-03 |
| 161 | miR-4521 | 1.75 | Up | 3.63 | -2.04 | 2.77E-03 |
| 162 | miR-944 | -1.76 | Down | -4.09 | -1.57 | 2.79E-03 |
| 163 | miR-493-3p | -1.08 | Down | -3.62 | -2.05 | 2.82E-03 |
| 164 | miR-1304-5p | 3.26 | Up | 3.60 | -2.08 | 2.91E-03 |
| 165 | miR-487b-3p | -1.14 | Down | -3.59 | -2.11 | 3.00E-03 |
| 166 | miR-676-3p | 1.66 | Up | 3.58 | -2.13 | 3.04E-03 |
| 167 | miR-653-5p | -2.47 | Down | -3.54 | -2.21 | 3.30E-03 |
| 168 | miR-376c-3p | -1.10 | Down | -3.53 | -2.23 | 3.39E-03 |
| 169 | miR-3200-3p | 1.05 | Up | 3.52 | -2.24 | 3.42E-03 |
| 170 | miR-4724-5p | 3.85 | Up | 3.71 | -2.01 | 3.49E-03 |
| 171 | miR-4777-3p | -2.16 | Down | -3.55 | -2.22 | 3.59E-03 |
| 172 | miR-337-5p | -1.19 | Down | -3.49 | -2.30 | 3.61E-03 |
| 173 | miR-150-5p | -1.87 | Down | -3.48 | -2.33 | 3.73E-03 |
| 174 | miR-3943 | -2.57 | Down | -3.65 | -2.14 | 3.87E-03 |
| 175 | miR-30b-5p | 1.11 | Up | 3.45 | -2.39 | 3.96E-03 |
| 176 | miR-4649-3p | -2.28 | Down | -3.82 | -1.98 | 4.15E-03 |
| 177 | miR-514b-3p | -4.64 | Down | -3.47 | -2.38 | 4.20E-03 |
| 178 | miR-1252-5p | 1.86 | Up | 3.40 | -2.48 | 4.35E-03 |
| 179 | miR-497-3p | 1.36 | Up | 3.40 | -2.49 | 4.39E-03 |
| 180 | miR-181a-3p | -1.08 | Down | -3.38 | -2.52 | 4.50E-03 |
| 181 | miR-139-5p | -1.12 | Down | -3.38 | -2.53 | 4.55E-03 |
| 182 | miR-6730-5p | -1.79 | Down | -3.36 | -2.55 | 4.68E-03 |
| 183 | miR-513b-5p | -3.08 | Down | -3.39 | -2.52 | 4.85E-03 |
| 184 | miR-126-5p | 2.86 | Up | 5.73 | -1.43 | 4.95E-03 |
| 185 | miR-33a-5p | 1.08 | Up | 3.33 | -2.61 | 4.97E-03 |
| 186 | miR-3653-5p | -1.24 | Down | -3.32 | -2.65 | 5.14E-03 |
| 187 | miR-3064-3p | -1.56 | Down | -3.31 | -2.66 | 5.22E-03 |
| 188 | miR-1322 | -1.46 | Down | -4.00 | -2.01 | 5.35E-03 |
| 189 | miR-4762-5p | 2.31 | Up | 3.38 | -2.58 | 5.56E-03 |
| 190 | miR-30b-3p | 1.04 | Up | 3.27 | -2.73 | 5.58E-03 |
| 191 | miR-549a | -1.85 | Down | -3.27 | -2.74 | 5.63E-03 |
| 192 | miR-1228-3p | -3.18 | Down | -3.25 | -2.77 | 5.85E-03 |
| 193 | miR-3129-5p | -1.54 | Down | -3.24 | -2.80 | 6.01E-03 |
| 194 | miR-551b-3p | -1.74 | Down | -3.23 | -2.80 | 6.04E-03 |
| 195 | miR-345-3p | 2.30 | Up | 3.21 | -2.86 | 6.38E-03 |
| 196 | miR-6087 | -1.68 | Down | -3.19 | -2.89 | 6.59E-03 |
| 197 | miR-376c-5p | -1.21 | Down | -3.19 | -2.89 | 6.59E-03 |
| 198 | miR-6818-5p | 1.26 | Up | 3.22 | -2.85 | 6.76E-03 |
| 199 | miR-3607-3p | -1.35 | Down | -3.16 | -2.95 | 6.99E-03 |
| 200 | miR-3614-5p | 1.58 | Up | 3.15 | -2.97 | 7.16E-03 |
| 201 | miR-142-5p | -1.14 | Down | -3.12 | -3.03 | 7.64E-03 |
| 202 | miR-491-5p | -1.16 | Down | -3.10 | -3.06 | 7.84E-03 |
| 203 | miR-4770 | -2.62 | Down | -3.09 | -3.09 | 8.10E-03 |
| 204 | let-7b-3p | -1.01 | Down | -3.08 | -3.10 | 8.16E-03 |
| 205 | miR-6503-5p | -1.32 | Down | -3.07 | -3.13 | 8.40E-03 |
| 206 | miR-146a-5p | -1.91 | Down | -3.04 | -3.17 | 8.80E-03 |
| 207 | miR-369-5p | -1.03 | Down | -3.04 | -3.19 | 8.96E-03 |
| 208 | miR-5695 | 1.46 | Up | 2.99 | -3.28 | 9.81E-03 |
| 209 | miR-376b-5p | -1.21 | Down | -2.97 | -3.31 | 1.01E-02 |
| 210 | miR-506-3p | -2.93 | Down | -3.01 | -3.25 | 1.02E-02 |
| 211 | miR-6892-5p | 2.82 | Up | 3.03 | -3.20 | 1.05E-02 |
| 212 | miR-4746-5p | 3.03 | Up | 2.94 | -3.38 | 1.08E-02 |
| 213 | miR-1276 | 1.86 | Up | 2.93 | -3.40 | 1.11E-02 |
| 214 | miR-6891-5p | 3.61 | Up | 5.70 | -2.07 | 1.17E-02 |
| 215 | miR-6513-3p | -1.63 | Down | -3.21 | -3.04 | 1.26E-02 |
| 216 | miR-509-5p | -2.83 | Down | -2.85 | -3.55 | 1.30E-02 |
| 217 | miR-3154 | -1.39 | Down | -2.85 | -3.54 | 1.38E-02 |
| 218 | miR-378b | 2.48 | Up | 4.23 | -2.39 | 1.40E-02 |
| 219 | miR-19b-3p | 1.72 | Up | 2.97 | -3.32 | 1.58E-02 |
| 220 | miR-142-3p | -1.14 | Down | -2.73 | -3.78 | 1.65E-02 |
| 221 | miR-4680-3p | -1.03 | Down | -2.88 | -3.51 | 1.65E-02 |
| 222 | miR-29a-5p | 1.20 | Up | 2.69 | -3.84 | 1.76E-02 |
| 223 | miR-377-5p | -1.23 | Down | -2.68 | -3.87 | 1.81E-02 |
| 224 | miR-137 | -2.48 | Down | -2.68 | -3.87 | 1.81E-02 |
| 225 | miR-7704 | -1.23 | Down | -2.66 | -3.90 | 1.86E-02 |
| 226 | miR-4679 | -2.93 | Down | -2.69 | -3.84 | 1.87E-02 |
| 227 | miR-191-5p | 1.15 | Up | 2.64 | -3.95 | 1.96E-02 |
| 228 | miR-766-3p | -1.04 | Down | -2.62 | -3.97 | 2.02E-02 |
| 229 | miR-4802-5p | -1.08 | Down | -2.68 | -3.85 | 2.03E-02 |
| 230 | miR-6838-5p | -2.68 | Down | -2.76 | -3.70 | 2.03E-02 |
| 231 | miR-1224-5p | 3.06 | Up | 2.79 | -3.61 | 2.11E-02 |
| 232 | miR-33b-5p | 1.20 | Up | 2.59 | -4.02 | 2.13E-02 |
| 233 | miR-605-3p | -2.13 | Down | -2.59 | -4.03 | 2.15E-02 |
| 234 | miR-3616-3p | 2.36 | Up | 2.95 | -3.21 | 2.19E-02 |
| 235 | miR-4473 | 1.26 | Up | 2.60 | -4.01 | 2.22E-02 |
| 236 | miR-6504-5p | -1.33 | Down | -2.64 | -3.88 | 2.30E-02 |
| 237 | miR-4670-3p | -1.30 | Down | -2.52 | -4.16 | 2.46E-02 |
| 238 | let-7d-3p | -1.23 | Down | -2.51 | -4.18 | 2.50E-02 |
| 239 | miR-4662b | -2.46 | Down | -2.48 | -4.23 | 2.80E-02 |
| 240 | miR-4753-5p | 1.20 | Up | 2.87 | -3.55 | 2.88E-02 |
| 241 | miR-5581-3p | 2.25 | Up | 2.46 | -4.25 | 2.88E-02 |
| 242 | miR-6743-3p | -1.48 | Down | -2.42 | -4.34 | 2.97E-02 |
| 243 | miR-3691-5p | 1.23 | Up | 2.42 | -4.34 | 2.98E-02 |
| 244 | miR-489-3p | -1.24 | Down | -2.41 | -4.35 | 3.01E-02 |
| 245 | miR-1291 | -1.10 | Down | -2.41 | -4.36 | 3.02E-02 |
| 246 | miR-6810-5p | -1.98 | Down | -2.39 | -4.39 | 3.15E-02 |
| 247 | miR-6772-3p | -3.51 | Down | -2.54 | -4.06 | 3.18E-02 |
| 248 | miR-205-5p | 2.48 | Up | 2.39 | -4.40 | 3.18E-02 |
| 249 | miR-488-5p | -1.47 | Down | -2.40 | -4.36 | 3.23E-02 |
| 250 | miR-1249-3p | -1.60 | Down | -2.38 | -4.40 | 3.35E-02 |
| 251 | miR-26a-1-3p | 1.86 | Up | 2.35 | -4.47 | 3.41E-02 |
| 252 | miR-6895-3p | 1.48 | Up | 3.16 | -3.40 | 3.53E-02 |
| 253 | miR-4802-3p | -1.00 | Down | -2.33 | -4.51 | 3.56E-02 |
| 254 | miR-372-3p | 1.46 | Up | 2.37 | -4.39 | 3.57E-02 |
| 255 | miR-371a-5p | 1.52 | Up | 2.31 | -4.54 | 3.69E-02 |
| 256 | miR-5579-3p | -2.28 | Down | -2.30 | -4.55 | 3.73E-02 |
| 257 | miR-3667-5p | 2.68 | Up | 3.62 | -3.17 | 3.82E-02 |
| 258 | miR-4664-3p | 2.73 | Up | 2.31 | -4.48 | 3.94E-02 |
| 259 | miR-6125 | -1.64 | Down | -2.27 | -4.59 | 4.12E-02 |
| 260 | miR-3614-3p | 1.18 | Up | 2.23 | -4.68 | 4.29E-02 |
| 261 | miR-450b-5p | 1.00 | Up | 2.22 | -4.69 | 4.34E-02 |
| 262 | miR-1245b-3p | 2.46 | Up | 2.27 | -4.54 | 4.47E-02 |
| 263 | miR-6766-3p | -1.64 | Down | -2.19 | -4.72 | 4.71E-02 |
