# Supplementary material for: Comprehensive characterization of endometrial competing endogenous RNA network in infertile women of childbearing age
Source: Aging (Albany NY). 2020 Feb 29;12(5):4204–21. doi: 10.18632/aging.102874 (PMC7093184; doi:10.18632/aging.102874)
Supplement: Supplementary Table 3 [file aging-12-102874-s002..docx]

**Supplementary Table 3. Full list of differentially expressed lncRNAs in Ectopic Endometrium.**

| **ID** | **Gene_Symbol** | **log_2_(Fold Change)** | **Regulation** | **t Value** | **B Value** | **P Value** |
| --- | --- | --- | --- | --- | --- | --- |
| 1 | FAM182A | 1.49 | Up | 2.99 | -3.13 | 8.08E-03 |
| 2 | H19 | -1.81 | Down | -6.29 | 3.86 | 6.92E-06 |
| 3 | LINC01116 | 3.62 | Up | 10.82 | 11.44 | 3.29E-09 |
| 4 | MIR4435-2HG | 1.43 | Up | 3.55 | -1.94 | 2.37E-03 |
| 5 | LRRC75A-AS1 | 1.00 | Up | 4.73 | 0.62 | 1.77E-04 |
| 6 | LINC01106 | -1.46 | Down | -6.29 | 3.85 | 6.97E-06 |
| 7 | C8orf31 | -1.88 | Down | -5.76 | 2.79 | 2.00E-05 |
| 8 | LINC00324 | 1.16 | Up | 5.07 | 1.34 | 8.60E-05 |
| 9 | OGFRP1 | -1.44 | Down | -3.42 | -2.22 | 3.14E-03 |
| 10 | SNHG28 | 3.42 | Up | 8.74 | 8.30 | 8.04E-08 |
| 11 | C9orf163 | -1.45 | Down | -4.71 | 0.58 | 1.84E-04 |
| 12 | MIRLET7BHG | -1.56 | Down | -5.02 | 1.24 | 9.50E-05 |
| 13 | AC011450.1 | 2.25 | Up | 6.01 | 3.30 | 1.21E-05 |
| 14 | AFDN-DT | -2.81 | Down | -8.45 | 7.81 | 1.32E-07 |
| 15 | C3orf35 | -1.67 | Down | -4.36 | -0.17 | 3.93E-04 |
| 16 | SNHG5 | 1.28 | Up | 4.16 | -0.62 | 6.15E-04 |
| 17 | LINC01123 | -2.42 | Down | -6.84 | 4.92 | 2.39E-06 |
| 18 | AP001043.1 | -2.82 | Down | -3.30 | -2.47 | 4.06E-03 |
| 19 | LOH12CR2 | 1.30 | Up | 5.21 | 1.65 | 6.29E-05 |
| 20 | Z99774.1 | 3.68 | Up | 5.51 | 2.28 | 3.36E-05 |
| 21 | AC016747.1 | 1.22 | Up | 5.05 | 1.30 | 8.93E-05 |
| 22 | UCA1 | -6.98 | Down | -9.45 | 9.42 | 2.58E-08 |
| 23 | LINC00887 | 1.58 | Up | 3.75 | -1.50 | 1.51E-03 |
| 24 | MEG3 | 1.54 | Up | 3.49 | -2.06 | 2.68E-03 |
| 25 | AC004540.1 | 2.76 | Up | 5.12 | 1.45 | 7.71E-05 |
| 26 | AL645728.1 | 1.26 | Up | 3.61 | -1.81 | 2.07E-03 |
| 27 | LINC02449 | -1.18 | Down | -3.63 | -1.76 | 1.97E-03 |
| 28 | MIR99AHG | 1.31 | Up | 3.72 | -1.56 | 1.61E-03 |
| 29 | MIR17HG | -1.34 | Down | -5.36 | 1.97 | 4.58E-05 |
| 30 | LINC00598 | -2.19 | Down | -3.46 | -2.14 | 2.89E-03 |
| 31 | LINC01139 | -1.56 | Down | -3.70 | -1.61 | 1.68E-03 |
| 32 | LINC01356 | 2.19 | Up | 7.15 | 5.50 | 1.33E-06 |
| 33 | CYTOR | 1.79 | Up | 3.96 | -1.06 | 9.62E-04 |
| 34 | AC005165.1 | -1.09 | Down | -2.82 | -3.46 | 1.15E-02 |
| 35 | LINC02593 | -2.58 | Down | -7.65 | 6.43 | 5.28E-07 |
| 36 | FAM95B1 | -1.82 | Down | -4.55 | 0.23 | 2.61E-04 |
| 37 | AL390729.1 | 2.51 | Up | 6.15 | 3.58 | 9.14E-06 |
| 38 | AL161785.1 | 1.23 | Up | 3.66 | -1.69 | 1.83E-03 |
| 39 | SMIM25 | 4.58 | Up | 8.41 | 7.75 | 1.41E-07 |
| 40 | AP001476.1 | 1.10 | Up | 2.28 | -4.52 | 3.56E-02 |
| 41 | LINC00240 | -1.28 | Down | -5.40 | 2.05 | 4.22E-05 |
| 42 | LINC00092 | -1.37 | Down | -2.55 | -4.01 | 2.05E-02 |
| 43 | LINC01678 | 2.39 | Up | 6.07 | 3.41 | 1.08E-05 |
| 44 | AC121247.1 | 1.53 | Up | 2.88 | -3.34 | 1.01E-02 |
| 45 | PTPRD-AS1 | 1.43 | Up | 2.81 | -3.49 | 1.18E-02 |
| 46 | MIAT | 3.41 | Up | 5.28 | 1.78 | 5.52E-05 |
| 47 | LINC00937 | -1.25 | Down | -3.07 | -2.95 | 6.68E-03 |
| 48 | LINC02519 | 2.44 | Up | 5.31 | 1.84 | 5.17E-05 |
| 49 | GAS1RR | 2.46 | Up | 5.05 | 1.31 | 8.88E-05 |
| 50 | HAGLROS | 2.32 | Up | 4.63 | 0.40 | 2.21E-04 |
| 51 | LINC-PINT | -1.33 | Down | -3.50 | -2.05 | 2.64E-03 |
| 52 | LINC02542 | 2.50 | Up | 7.86 | 6.79 | 3.66E-07 |
| 53 | TEX41 | 2.80 | Up | 6.38 | 4.03 | 5.82E-06 |
| 54 | AF127936.1 | 2.24 | Up | 5.38 | 2.01 | 4.38E-05 |
| 55 | AC109826.1 | 3.62 | Up | 7.09 | 5.40 | 1.48E-06 |
| 56 | AC005082.1 | -2.01 | Down | -5.66 | 2.57 | 2.50E-05 |
| 57 | AC244021.1 | 3.00 | Up | 5.87 | 3.02 | 1.59E-05 |
| 58 | AC017074.1 | -1.08 | Down | -2.32 | -4.45 | 3.29E-02 |
| 59 | LINC01806 | -2.01 | Down | -2.88 | -3.35 | 1.02E-02 |
| 60 | SLC2A1-AS1 | -2.00 | Down | -3.75 | -1.51 | 1.52E-03 |
| 61 | AC018647.1 | 1.67 | Up | 3.33 | -2.40 | 3.80E-03 |
| 62 | LINC01132 | -1.19 | Down | -3.11 | -2.87 | 6.16E-03 |
| 63 | AC239809.3 | 1.04 | Up | 4.41 | -0.07 | 3.53E-04 |
| 64 | LINC01341 | -1.72 | Down | -3.67 | -1.68 | 1.81E-03 |
| 65 | LINC01515 | 1.39 | Up | 3.27 | -2.53 | 4.34E-03 |
| 66 | LINC01320 | -5.89 | Down | -5.65 | 2.57 | 2.50E-05 |
| 67 | AC005013.1 | 2.89 | Up | 7.41 | 5.98 | 8.26E-07 |
| 68 | UBAC2-AS1 | -1.23 | Down | -3.54 | -1.96 | 2.42E-03 |
| 69 | AL137026.1 | 3.00 | Up | 11.40 | 12.21 | 1.48E-09 |
| 70 | LINC01715 | 1.54 | Up | 3.61 | -1.81 | 2.06E-03 |
| 71 | AJ009632.2 | 1.59 | Up | 3.97 | -1.03 | 9.38E-04 |
| 72 | U73166.1 | -1.76 | Down | -4.71 | 0.58 | 1.85E-04 |
| 73 | AC108463.2 | 1.20 | Up | 2.40 | -4.29 | 2.77E-02 |
| 74 | AC092162.2 | 1.78 | Up | 4.73 | 0.63 | 1.76E-04 |
| 75 | AL139220.2 | 3.43 | Up | 6.47 | 4.22 | 4.83E-06 |
| 76 | MANCR | 1.47 | Up | 2.90 | -3.30 | 9.71E-03 |
| 77 | SGO1-AS1 | -1.28 | Down | -2.77 | -3.56 | 1.27E-02 |
| 78 | LINC00689 | 1.75 | Up | 3.01 | -3.08 | 7.64E-03 |
| 79 | AL078590.2 | -2.15 | Down | -3.13 | -2.83 | 5.95E-03 |
| 80 | LINC01798 | -2.42 | Down | -4.88 | 0.94 | 1.29E-04 |
| 81 | AC105277.1 | 1.94 | Up | 7.95 | 6.96 | 3.11E-07 |
| 82 | AL136368.1 | 1.18 | Up | 2.42 | -4.25 | 2.66E-02 |
| 83 | LINC00327 | 1.28 | Up | 2.30 | -4.47 | 3.38E-02 |
| 84 | MIR3936HG | -2.06 | Down | -8.25 | 7.47 | 1.86E-07 |
| 85 | ERVH48-1 | -1.20 | Down | -2.90 | -3.29 | 9.61E-03 |
| 86 | LINC00702 | 2.87 | Up | 4.90 | 0.98 | 1.23E-04 |
| 87 | LINC00472 | -2.00 | Down | -7.64 | 6.41 | 5.37E-07 |
| 88 | AL590094.1 | 1.21 | Up | 3.74 | -1.53 | 1.55E-03 |
| 89 | LINC01503 | 1.48 | Up | 4.45 | 0.01 | 3.27E-04 |
| 90 | AC106869.1 | -1.22 | Down | -3.40 | -2.27 | 3.30E-03 |
| 91 | AL136309.2 | -1.26 | Down | -2.85 | -3.40 | 1.08E-02 |
| 92 | MIR155HG | 1.36 | Up | 3.60 | -1.83 | 2.10E-03 |
| 93 | LINC00957 | 1.98 | Up | 7.17 | 5.55 | 1.27E-06 |
| 94 | AC073046.1 | -1.69 | Down | -4.91 | 1.00 | 1.20E-04 |
| 95 | LINC00607 | 3.53 | Up | 5.81 | 2.89 | 1.83E-05 |
| 96 | LINC00941 | 2.52 | Up | 5.66 | 2.57 | 2.50E-05 |
| 97 | LINC01814 | -1.49 | Down | -7.74 | 6.58 | 4.55E-07 |
| 98 | AC110995.1 | 3.39 | Up | 6.75 | 4.75 | 2.83E-06 |
| 99 | KCCAT333 | -6.58 | Down | -11.21 | 11.97 | 1.90E-09 |
| 100 | AC024560.1 | -2.14 | Down | -3.95 | -1.08 | 9.83E-04 |
| 101 | LINC01554 | 1.12 | Up | 2.35 | -4.38 | 3.07E-02 |
| 102 | LINC01852 | 1.80 | Up | 4.53 | 0.19 | 2.71E-04 |
| 103 | ZNF295-AS1 | -2.27 | Down | -3.85 | -1.29 | 1.22E-03 |
| 104 | LINC01502 | -5.80 | Down | -4.17 | -0.59 | 5.95E-04 |
| 105 | LINC01637 | 1.86 | Up | 5.48 | 2.20 | 3.62E-05 |
| 106 | LINC01684 | -1.53 | Down | -3.60 | -1.83 | 2.10E-03 |
| 107 | AC007384.1 | 1.60 | Up | 3.78 | -1.44 | 1.41E-03 |
| 108 | AC022034.1 | 3.59 | Up | 4.98 | 1.15 | 1.04E-04 |
| 109 | LINC01238 | -1.98 | Down | -3.71 | -1.59 | 1.65E-03 |
| 110 | LINC01679 | 1.85 | Up | 4.05 | -0.84 | 7.73E-04 |
| 111 | AC244197.2 | -1.73 | Down | -3.02 | -3.06 | 7.50E-03 |
| 112 | AC092691.1 | -2.31 | Down | -2.93 | -3.24 | 9.06E-03 |
| 113 | LINC01119 | 1.74 | Up | 4.59 | 0.33 | 2.37E-04 |
| 114 | AC007405.3 | -2.68 | Down | -9.00 | 8.71 | 5.29E-08 |
| 115 | AL512306.2 | -1.31 | Down | -2.65 | -3.81 | 1.65E-02 |
| 116 | LINC00877 | -1.37 | Down | -3.37 | -2.32 | 3.49E-03 |
| 117 | LINC00996 | -1.24 | Down | -2.58 | -3.95 | 1.93E-02 |
| 118 | AC108693.2 | -1.09 | Down | -2.70 | -3.71 | 1.48E-02 |
| 119 | LINC00882 | 1.85 | Up | 4.41 | -0.07 | 3.55E-04 |
| 120 | DUBR | 1.53 | Up | 6.10 | 3.48 | 1.01E-05 |
| 121 | AC147067.1 | 2.04 | Up | 6.44 | 4.14 | 5.20E-06 |
| 122 | LINC00847 | 1.14 | Up | 6.30 | 3.87 | 6.82E-06 |
| 123 | LINC00861 | 2.56 | Up | 4.92 | 1.03 | 1.17E-04 |
| 124 | LINC00461 | -1.04 | Down | -2.20 | -4.66 | 4.15E-02 |
| 125 | CRNDE | -1.45 | Down | -5.19 | 1.59 | 6.66E-05 |
| 126 | DRAIC | -4.31 | Down | -5.78 | 2.83 | 1.94E-05 |
| 127 | SNHG6 | 1.21 | Up | 6.76 | 4.76 | 2.79E-06 |
| 128 | LINC02273 | 3.16 | Up | 4.81 | 0.80 | 1.47E-04 |
| 129 | LINC01550 | 2.51 | Up | 5.89 | 3.05 | 1.55E-05 |
| 130 | AC090510.1 | 1.03 | Up | 5.59 | 2.44 | 2.85E-05 |
| 131 | LINC02288 | -1.40 | Down | -2.57 | -3.96 | 1.95E-02 |
| 132 | PICART1 | -1.40 | Down | -2.14 | -4.77 | 4.68E-02 |
| 133 | LINC00920 | 1.38 | Up | 4.83 | 0.84 | 1.42E-04 |
| 134 | MIR210HG | -2.18 | Down | -5.10 | 1.41 | 8.00E-05 |
| 135 | PCED1B-AS1 | 1.59 | Up | 3.47 | -2.11 | 2.83E-03 |
| 136 | LINC02268 | -2.12 | Down | -4.70 | 0.55 | 1.90E-04 |
| 137 | LINC01197 | 2.40 | Up | 8.01 | 7.06 | 2.80E-07 |
| 138 | AC002070.1 | -1.17 | Down | -5.25 | 1.72 | 5.86E-05 |
| 139 | LINC02432 | -1.95 | Down | -2.13 | -4.78 | 4.75E-02 |
| 140 | LINC02362 | 2.56 | Up | 3.94 | -1.09 | 9.89E-04 |
| 141 | CARMN | 1.43 | Up | 3.31 | -2.45 | 4.00E-03 |
| 142 | AC126768.3 | -1.05 | Down | -4.67 | 0.48 | 2.03E-04 |
| 143 | AC092490.1 | -2.08 | Down | -2.79 | -3.54 | 1.24E-02 |
| 144 | LINC02021 | 1.76 | Up | 3.63 | -1.77 | 1.99E-03 |
| 145 | FZD10-DT | -1.68 | Down | -3.27 | -2.52 | 4.31E-03 |
| 146 | AP002884.1 | 2.71 | Up | 8.79 | 8.37 | 7.50E-08 |
| 147 | CXXC5-AS1 | -1.22 | Down | -2.80 | -3.51 | 1.21E-02 |
| 148 | LINC02381 | 5.79 | Up | 13.58 | 14.87 | 9.13E-11 |
| 149 | LINC02600 | -4.00 | Down | -5.92 | 3.12 | 1.45E-05 |
| 150 | AC244502.1 | -1.66 | Down | -2.72 | -3.67 | 1.42E-02 |
| 151 | LINC00923 | -1.57 | Down | -5.21 | 1.64 | 6.38E-05 |
| 152 | AP003086.1 | -1.12 | Down | -2.62 | -3.86 | 1.75E-02 |
| 153 | LINC01301 | -1.07 | Down | -2.48 | -4.15 | 2.37E-02 |
| 154 | AC145285.2 | -1.12 | Down | -3.48 | -2.08 | 2.73E-03 |
| 155 | LINC01094 | 3.40 | Up | 6.41 | 4.09 | 5.46E-06 |
| 156 | LINC01605 | 1.56 | Up | 3.52 | -1.99 | 2.50E-03 |
| 157 | MIR3142HG | 1.78 | Up | 2.43 | -4.23 | 2.60E-02 |
| 158 | MAL2-AS1 | -5.60 | Down | -6.92 | 5.07 | 2.05E-06 |
| 159 | AC087273.2 | -1.87 | Down | -2.48 | -4.13 | 2.33E-02 |
| 160 | AC009812.3 | -1.98 | Down | -6.09 | 3.46 | 1.03E-05 |
| 161 | FLJ20021 | 1.28 | Up | 5.67 | 2.60 | 2.43E-05 |
| 162 | AC027031.2 | -1.37 | Down | -4.28 | -0.36 | 4.72E-04 |
| 163 | AC022182.1 | 2.16 | Up | 4.88 | 0.94 | 1.28E-04 |
| 164 | AC022182.2 | 2.08 | Up | 3.98 | -1.01 | 9.12E-04 |
| 165 | AP001189.3 | 1.30 | Up | 2.35 | -4.39 | 3.07E-02 |
| 166 | MIR100HG | 1.90 | Up | 6.51 | 4.28 | 4.52E-06 |
| 167 | AP004608.1 | -6.16 | Down | -8.13 | 7.26 | 2.29E-07 |
| 168 | LINC00346 | -1.19 | Down | -3.44 | -2.17 | 2.98E-03 |
| 169 | ADGRD1-AS1 | -1.76 | Down | -5.24 | 1.71 | 5.92E-05 |
| 170 | AC027290.1 | -1.50 | Down | -6.91 | 5.06 | 2.07E-06 |
| 171 | AC026333.3 | 2.27 | Up | 4.12 | -0.71 | 6.73E-04 |
| 172 | AC007406.3 | 1.38 | Up | 2.76 | -3.60 | 1.32E-02 |
| 173 | LINC02298 | -1.40 | Down | -4.28 | -0.36 | 4.74E-04 |
| 174 | AC027288.3 | 1.59 | Up | 2.51 | -4.08 | 2.22E-02 |
| 175 | AC005871.2 | -1.37 | Down | -6.24 | 3.75 | 7.67E-06 |
| 176 | VASH1-AS1 | 1.21 | Up | 5.88 | 3.04 | 1.57E-05 |
| 177 | AC013451.1 | 5.58 | Up | 9.73 | 9.86 | 1.66E-08 |
| 178 | AL157871.1 | -1.91 | Down | -6.70 | 4.65 | 3.13E-06 |
| 179 | AL359317.1 | 1.69 | Up | 3.50 | -2.04 | 2.62E-03 |
| 180 | AC007376.2 | 1.01 | Up | 2.20 | -4.65 | 4.13E-02 |
| 181 | LINC01269 | 1.47 | Up | 2.69 | -3.73 | 1.52E-02 |
| 182 | AL358334.2 | -2.16 | Down | -3.42 | -2.21 | 3.12E-03 |
| 183 | LINC02328 | 1.08 | Up | 2.43 | -4.23 | 2.61E-02 |
| 184 | LINC01579 | -1.52 | Down | -2.39 | -4.32 | 2.85E-02 |
| 185 | LINC02289 | -1.80 | Down | -3.60 | -1.83 | 2.12E-03 |
| 186 | AL135818.1 | 2.45 | Up | 3.73 | -1.56 | 1.60E-03 |
| 187 | TGFB3-AS1 | 1.48 | Up | 4.17 | -0.59 | 5.99E-04 |
| 188 | AL355075.3 | -1.06 | Down | -2.90 | -3.31 | 9.76E-03 |
| 189 | LINC00924 | 2.07 | Up | 4.18 | -0.58 | 5.91E-04 |
| 190 | AC087477.5 | -1.24 | Down | -2.42 | -4.26 | 2.68E-02 |
| 191 | CERNA1 | -1.90 | Down | -8.07 | 7.16 | 2.53E-07 |
| 192 | AC084757.3 | 1.86 | Up | 3.39 | -2.27 | 3.33E-03 |
| 193 | AC022087.1 | -1.12 | Down | -3.11 | -2.87 | 6.15E-03 |
| 194 | AC009093.1 | 3.75 | Up | 8.70 | 8.23 | 8.66E-08 |
| 195 | AC083843.2 | -1.31 | Down | -3.55 | -1.93 | 2.34E-03 |
| 196 | AL365361.1 | 3.59 | Up | 6.58 | 4.42 | 3.94E-06 |
| 197 | SH3RF3-AS1 | 1.01 | Up | 3.77 | -1.46 | 1.45E-03 |
| 198 | AC009414.2 | 1.70 | Up | 3.75 | -1.51 | 1.52E-03 |
| 199 | NORAD | 1.09 | Up | 3.83 | -1.33 | 1.27E-03 |
| 200 | AC141586.2 | -1.28 | Down | -2.84 | -3.42 | 1.10E-02 |
| 201 | LINC02166 | -1.76 | Down | -7.16 | 5.52 | 1.31E-06 |
| 202 | AC009812.4 | -1.37 | Down | -2.94 | -3.22 | 8.90E-03 |
| 203 | AL023284.4 | -2.63 | Down | -6.32 | 3.92 | 6.49E-06 |
| 204 | LINC02367 | -2.54 | Down | -6.56 | 4.40 | 4.04E-06 |
| 205 | NBAT1 | -1.55 | Down | -4.71 | 0.57 | 1.85E-04 |
| 206 | AL590787.1 | 1.09 | Up | 2.27 | -4.54 | 3.63E-02 |
| 207 | AC009093.2 | 3.40 | Up | 10.59 | 11.12 | 4.59E-09 |
| 208 | AC027702.1 | 1.43 | Up | 3.96 | -1.05 | 9.51E-04 |
| 209 | LINC01541 | -7.34 | Down | -10.54 | 11.04 | 4.95E-09 |
| 210 | AC010542.2 | 1.10 | Up | 4.63 | 0.40 | 2.21E-04 |
| 211 | AC073657.1 | -1.03 | Down | -2.79 | -3.53 | 1.23E-02 |
| 212 | AL591848.4 | -1.51 | Down | -2.94 | -3.23 | 8.98E-03 |
| 213 | LINC00565 | 1.89 | Up | 4.46 | 0.04 | 3.17E-04 |
| 214 | LINC02516 | 1.04 | Up | 2.99 | -3.12 | 8.00E-03 |
| 215 | RBFADN | -1.25 | Down | -3.80 | -1.40 | 1.36E-03 |
| 216 | AL031123.2 | -1.76 | Down | -4.49 | 0.11 | 2.96E-04 |
| 217 | AC009061.1 | -3.81 | Down | -5.54 | 2.34 | 3.16E-05 |
| 218 | LINC01686 | 1.50 | Up | 4.04 | -0.88 | 8.00E-04 |
| 219 | AL359694.2 | 2.37 | Up | 5.11 | 1.43 | 7.86E-05 |
| 220 | AC144831.1 | 1.17 | Up | 3.95 | -1.06 | 9.66E-04 |
| 221 | AC108134.2 | -1.17 | Down | -3.02 | -3.05 | 7.47E-03 |
| 222 | DKFZP434A062 | 1.12 | Up | 3.50 | -2.04 | 2.62E-03 |
| 223 | AC005736.1 | 1.34 | Up | 2.85 | -3.41 | 1.09E-02 |
| 224 | AC108134.3 | 2.43 | Up | 5.97 | 3.22 | 1.31E-05 |
| 225 | AC007114.1 | -1.28 | Down | -3.27 | -2.54 | 4.37E-03 |
| 226 | AL445686.2 | -1.24 | Down | -4.10 | -0.74 | 6.95E-04 |
| 227 | AP000915.1 | 3.37 | Up | 7.96 | 6.96 | 3.08E-07 |
| 228 | PRR29-AS1 | -3.07 | Down | -7.54 | 6.22 | 6.53E-07 |
| 229 | AC005899.3 | 1.03 | Up | 2.49 | -4.12 | 2.31E-02 |
| 230 | AC138207.5 | 2.58 | Up | 4.30 | -0.32 | 4.53E-04 |
| 231 | LINC01927 | -1.52 | Down | -2.94 | -3.22 | 8.89E-03 |
| 232 | AC005838.2 | -1.38 | Down | -3.22 | -2.64 | 4.86E-03 |
| 233 | AC093484.4 | -1.35 | Down | -3.47 | -2.10 | 2.78E-03 |
| 234 | AC061992.1 | 1.51 | Up | 2.55 | -4.00 | 2.02E-02 |
| 235 | LINC01140 | 1.71 | Up | 4.93 | 1.05 | 1.14E-04 |
| 236 | LINC01764 | -6.67 | Down | -9.90 | 10.11 | 1.29E-08 |
| 237 | AC022706.1 | 2.24 | Up | 3.83 | -1.33 | 1.27E-03 |
| 238 | ZNF582-AS1 | 1.40 | Up | 3.40 | -2.26 | 3.29E-03 |
| 239 | AC021683.1 | -1.67 | Down | -3.65 | -1.73 | 1.90E-03 |
| 240 | AC021683.2 | -1.95 | Down | -4.81 | 0.80 | 1.48E-04 |
| 241 | AC243960.1 | 1.40 | Up | 2.67 | -3.77 | 1.58E-02 |
| 242 | AC092279.1 | -1.29 | Down | -4.04 | -0.87 | 7.90E-04 |
| 243 | FENDRR | -1.79 | Down | -4.11 | -0.73 | 6.88E-04 |
| 244 | AC010329.1 | -2.24 | Down | -2.86 | -3.38 | 1.05E-02 |
| 245 | AL009178.2 | 1.57 | Up | 2.73 | -3.64 | 1.39E-02 |
| 246 | AC010487.2 | -3.65 | Down | -7.98 | 7.00 | 2.98E-07 |
| 247 | AL513318.2 | 1.48 | Up | 2.92 | -3.26 | 9.23E-03 |
| 248 | AC012065.4 | -5.08 | Down | -10.12 | 10.44 | 9.17E-09 |
| 249 | AC104109.2 | -1.87 | Down | -4.71 | 0.58 | 1.84E-04 |
| 250 | SMC2-AS1 | -1.12 | Down | -4.09 | -0.76 | 7.11E-04 |
| 251 | AC026367.1 | -1.89 | Down | -5.58 | 2.41 | 2.94E-05 |
| 252 | LINC01235 | 3.27 | Up | 5.28 | 1.78 | 5.50E-05 |
| 253 | AC116667.1 | 1.15 | Up | 3.21 | -2.65 | 4.91E-03 |
| 254 | AC026412.3 | -1.53 | Down | -5.71 | 2.69 | 2.23E-05 |
| 255 | AC016737.1 | -1.19 | Down | -4.07 | -0.81 | 7.45E-04 |
| 256 | LINC02550 | 2.51 | Up | 6.18 | 3.64 | 8.60E-06 |
| 257 | AL080317.2 | -1.11 | Down | -3.15 | -2.79 | 5.70E-03 |
| 258 | AP001330.5 | 1.33 | Up | 4.55 | 0.22 | 2.64E-04 |
| 259 | AL365181.2 | -1.67 | Down | -3.55 | -1.95 | 2.38E-03 |
| 260 | AL133406.3 | -1.50 | Down | -3.46 | -2.13 | 2.87E-03 |
| 261 | AC011374.2 | 1.31 | Up | 3.00 | -3.10 | 7.82E-03 |
| 262 | AC092687.3 | -4.76 | Down | -9.89 | 10.09 | 1.31E-08 |
| 263 | AP002360.3 | -1.05 | Down | -3.43 | -2.18 | 3.03E-03 |
| 264 | AC008035.1 | -1.34 | Down | -3.21 | -2.65 | 4.93E-03 |
| 265 | AL356056.3 | 1.70 | Up | 5.08 | 1.36 | 8.40E-05 |
| 266 | U62317.1 | 2.80 | Up | 4.91 | 1.00 | 1.20E-04 |
| 267 | GAS6-DT | 1.70 | Up | 5.55 | 2.35 | 3.13E-05 |
| 268 | AL022322.1 | -1.17 | Down | -2.99 | -3.11 | 7.95E-03 |
| 269 | AC004982.1 | -2.22 | Down | -6.37 | 4.01 | 5.95E-06 |
| 270 | AC245297.2 | 1.85 | Up | 4.85 | 0.88 | 1.36E-04 |
| 271 | AC015712.6 | -1.16 | Down | -2.90 | -3.29 | 9.61E-03 |
| 272 | AC004982.2 | -2.95 | Down | -6.78 | 4.82 | 2.65E-06 |
| 273 | AL353708.3 | 1.06 | Up | 4.50 | 0.12 | 2.92E-04 |
| 274 | AC107294.2 | 1.97 | Up | 5.35 | 1.93 | 4.74E-05 |
| 275 | AL117339.4 | 1.25 | Up | 2.82 | -3.47 | 1.15E-02 |
| 276 | AL078644.1 | -1.70 | Down | -3.23 | -2.61 | 4.70E-03 |
| 277 | AC007663.3 | -1.06 | Down | -2.78 | -3.54 | 1.25E-02 |
| 278 | AL035563.1 | 1.53 | Up | 5.07 | 1.36 | 8.44E-05 |
| 279 | AC006946.2 | 2.01 | Up | 3.92 | -1.13 | 1.03E-03 |
| 280 | AC137630.4 | -1.23 | Down | -2.63 | -3.84 | 1.71E-02 |
| 281 | AC069200.1 | -2.34 | Down | -5.03 | 1.25 | 9.35E-05 |
| 282 | AC009237.15 | -1.45 | Down | -3.72 | -1.58 | 1.63E-03 |
| 283 | LINC02019 | 1.26 | Up | 2.44 | -4.22 | 2.56E-02 |
| 284 | AC015819.1 | 1.67 | Up | 5.82 | 2.91 | 1.78E-05 |
| 285 | AL035661.1 | -3.08 | Down | -5.84 | 2.95 | 1.72E-05 |
| 286 | AC015912.3 | -1.86 | Down | -7.06 | 5.34 | 1.58E-06 |
| 287 | AC245297.3 | 1.15 | Up | 4.18 | -0.57 | 5.84E-04 |
| 288 | AL355338.1 | -1.04 | Down | -2.65 | -3.80 | 1.65E-02 |
| 289 | AL136964.1 | -1.26 | Down | -2.58 | -3.95 | 1.93E-02 |
| 290 | AC091982.3 | 1.09 | Up | 3.40 | -2.25 | 3.25E-03 |
| 291 | AC073534.1 | -1.26 | Down | -3.32 | -2.43 | 3.89E-03 |
| 292 | AC239799.2 | 1.08 | Up | 2.45 | -4.19 | 2.48E-02 |
| 293 | AC116407.2 | 1.28 | Up | 4.54 | 0.21 | 2.66E-04 |
| 294 | AC018553.1 | -1.87 | Down | -4.27 | -0.38 | 4.84E-04 |
| 295 | PGM5P3-AS1 | -4.17 | Down | -6.55 | 4.37 | 4.17E-06 |
| 296 | AL138995.1 | 1.17 | Up | 4.01 | -0.95 | 8.60E-04 |
| 297 | AL138478.1 | 1.21 | Up | 4.86 | 0.89 | 1.34E-04 |
| 298 | AL161669.3 | -1.84 | Down | -4.77 | 0.71 | 1.61E-04 |
| 299 | AC099518.6 | 1.08 | Up | 2.86 | -3.40 | 1.07E-02 |
| 300 | AC000403.1 | 1.47 | Up | 5.55 | 2.35 | 3.12E-05 |
| 301 | AC005332.7 | 1.09 | Up | 2.61 | -3.89 | 1.80E-02 |
| 302 | AC026401.3 | -1.08 | Down | -4.45 | 0.01 | 3.27E-04 |
| 303 | ERICD | -1.38 | Down | -2.83 | -3.44 | 1.12E-02 |
| 304 | PCAT14 | -2.11 | Down | -3.57 | -1.88 | 2.23E-03 |
| 305 | LINC01943 | 1.57 | Up | 3.96 | -1.04 | 9.47E-04 |
| 306 | LINC01232 | 1.67 | Up | 7.77 | 6.64 | 4.27E-07 |
| 307 | AC007878.1 | -1.10 | Down | -3.88 | -1.22 | 1.13E-03 |
| 308 | LINC00891 | 4.79 | Up | 9.68 | 9.78 | 1.79E-08 |
| 309 | AC092807.3 | 1.89 | Up | 4.25 | -0.41 | 5.01E-04 |
| 310 | AC215522.2 | 1.69 | Up | 3.99 | -0.99 | 8.96E-04 |
| 311 | BISPR | 2.73 | Up | 9.00 | 8.71 | 5.33E-08 |
| 312 | AL627171.2 | 1.19 | Up | 3.44 | -2.17 | 2.99E-03 |
| 313 | AL137077.2 | -1.44 | Down | -4.90 | 0.99 | 1.22E-04 |
